# Random capillary glucose levels throughout pregnancy, obstetric and neonatal outcomes, and long-term neurodevelopmental conditions in children: a group-based trajectory analysis

**DOI:** 10.1186/s12916-023-02926-3

**Published:** 2023-07-19

**Authors:** Shuyun Chen, Margareta Persson, Rui Wang, Christina Dalman, Brian K. Lee, Håkan Karlsson, Renee M. Gardner

**Affiliations:** 1 Department of Global Public Health, Karolinska Institutet, Stockholm, Sweden; 2 Department of Nursing, Umeå University, Umeå, Sweden; 3 The Swedish School of Sport and Health Sciences, GIH, Stockholm, Sweden; 4 Department of Neurobiology, Care Sciences and Society (NVS), Karolinska Institutet, Stockholm, Sweden; 5 Department of Epidemiology and Biostatistics, Drexel University School of Public Health, Philadelphia, PA USA; 6 A.J. Drexel Autism Institute, Philadelphia, PA USA; 7 Department of Neuroscience, Karolinska Institutet, Stockholm, Sweden

**Keywords:** Maternal glucose levels, Obstetric outcomes, Neonatal outcomes, Autism, Intellectual disability, Attention-deficit/hyperactivity disorder

## Abstract

**Background:**

Gestational diabetes mellitus (GDM) is associated with both short- and long-term risks, although it is unknown if risks vary by severity, timing, and duration of gestational hyperglycemia. We aimed to identify trajectories of random capillary glucose (RCG) levels throughout pregnancy and assess their associations with both obstetric/neonatal outcomes and children’s risk of neurodevelopmental conditions (NDCs) (i.e., autism, intellectual disability, and attention-deficit/hyperactivity disorders [ADHD]).

**Methods:**

A population-based cohort study was conducted involving 76,228 children born to 68,768 mothers without pregestational diabetes. Group-based trajectory modeling was utilized to identify distinct glucose trajectories across RCG values throughout the course of pregnancy. The associations between these trajectory groups and obstetric/neonatal outcomes as well as children’s NDCs were then assessed using generalized estimating equation models with a logit link. The Benjamini-Hochberg (BH) procedure was employed to adjust *P*-values for multiple comparisons, controlling the false discovery rate (FDR).

**Results:**

Five distinct glucose trajectory groups were identified, each with varying percentages diagnosed with GDM. Their associations with obstetric/neonatal outcomes as well as children’s NDCs varied. For example, when compared to the “Persistently Low” group, other groups exhibited varying degrees of increased risk for large-for-gestational-age babies, with the exception of the “High in Early Pregnancy” group. Compared to the “Persistently Low” group, all other trajectory groups were associated with NDC outcomes, except the “High in Mid-Pregnancy” group. However, none of the associations with offspring NDCs remained significant after accounting for the FDR correction.

**Conclusions:**

Persistent high glucose levels or moderately elevated glucose levels throughout pregnancy, as well as transient states of hyperglycemia in early or mid-pregnancy, were found to be associated with increased risks of specific obstetric and neonatal complications, and potentially offspring NDCs. These risks varied depending on the severity, timing, duration, and management of hyperglycemia. The findings underscore the need for continuous surveillance and individualized management strategies for women displaying different glucose trajectories during pregnancy. Limitations such as potential residual confounding, the role of mediators, and small sample size should be addressed in future studies.

**Supplementary Information:**

The online version contains supplementary material available at 10.1186/s12916-023-02926-3.

## Background

Hyperglycemia in pregnancy is associated with maternal overweight or obesity, which may complicate pregnancies, fetal health, and births [1, 2]. Hyperglycemia meeting the diagnostic criteria for gestational diabetes mellitus (GDM) has been associated with obstetric/neonatal complications such as pre-eclampsia, preterm birth, abnormal birthweight, neonatal hypoxia, and neonatal hypoglycemia [3, 4], though the Hyperglycemia and Adverse Pregnancy Outcomes (HAPO) study established that even subclinical glucose levels (hyperglycemia not meeting criteria for diagnosis of GDM), measured around 28 weeks of gestation, are associated with short-term health risks [5]. Exposure to maternal GDM has been linked with increased likelihood of neurodevelopmental conditions (NDCs) in children including autism [6–11], intellectual disability (ID) [6, 9, 12], and attention-deficit/hyperactivity disorders (ADHD) [6, 13–15]. The magnitude of these risks is likely dependent on timing of exposure to GDM [6, 7]. While it is plausible that GDM could directly influence the developing central nervous system [16], associations between GDM and NDCs may also be mediated by obstetric/neonatal complications, which are also associated with increased risks of NDCs [17–21].

Studies investigating risks associated with maternal hyperglycemia, particularly those investigating long-term outcomes like NDC diagnoses, have relied primarily on diagnoses of GDM as an exposure without information on blood glucose measurements [6–11]. Previous studies have not been able to assess if better glycemic control after the diagnosis of GDM might influence the risk of NDC outcomes [7, 13]. Furthermore, previous studies investigating the associations with offspring NDCs have not been able to control directly for confounders such as maternal psychiatric history [7, 13]. By relying on diagnoses of GDM as an exposure, it is not possible to establish whether exposure to subclinical hyperglycemia may still be harmful to children’s neurodevelopment in the long term. Moreover, the criteria and screening schedule for diagnosing GDM vary across countries and regions [22], with potential discrepancies from the internationally recommended standard [5, 23]. Previous studies have shown that selective screening missed around one third of GDM cases compared to universal screening [24, 25]. The gestational week in which GDM is first detected is strongly dependent on the screening schedule used [26–28]. This may delay the identification of the true onset of GDM and potentially mask the identification of sensitive time periods during which exposure to hyperglycemia may have the greatest influence. Though effective glycemic control may prevent short-term perinatal complications [29, 30], it is unknown if a transient exposure to hyperglycemia accompanied by subsequent effective control may reduce the risk of NDCs in children compared to continuous exposure. Furthermore, it is unclear if hyperglycemic states in the low-risk population, without overt GDM diagnoses or risk factors such as overweight/obesity, are associated with perinatal complications or long-term NDCs in offspring.

In Sweden, there was no consensus on the GDM diagnostic criteria in Sweden during our study period [31]. Random capillary glucose (RCG) is routinely measured by fingertip blood at multiple antenatal visits throughout pregnancy [31]. Women with an RCG level above 8–9 mmol/l are subsequently referred for an OGTT to exclude GDM [31].

In this population-based cohort study, we first aimed to identify and characterize distinct trajectories of RCG levels throughout pregnancy using group-based trajectory modeling (GBTM). We examined the associations of maternal glucose trajectory groups with obstetric/neonatal complications and children’s risks of NDCs, to understand whether these relationships varied with regard to severity, duration, and timing of maternal hyperglycemia.

## Methods

### Study population

This population-based cohort study is nested within the larger Stockholm Youth Cohort (SYC), a longitudinal record-linkage study of children resident in Stockholm 2001–2011. We linked electronic antenatal care records for births in Stockholm (from the medical journal system Obstetrix [32]) available from January 1, 2007, to December 31, 2010, to other healthcare and administrative registers, including the Medical Birth Register (MBR), for individuals within the SYC [33].

Of the 88,470 children (76,731 mothers) with births recorded 2007–2010 in Obstetrix, 636 (0.72%) children had no maternal random capillary glucose (RCG) records. Children were followed up until December 31, 2016. We excluded children who were residents in Sweden for less than 5 years or had no information on the length of residence, from multiple births, diagnosed with both an NDC and congenital malformations or inborn errors of metabolism (under the assumption that these outcomes were due to the documented genetic condition), or exposed to maternal pregestational diabetes (Additional file 1, Fig S1). For each RCG record, we excluded extreme RCG recordings outside the range of 2.8–33.3 mmol/l (which are associated with acute clinical effects [34]), those without a date recorded at sampling, or those where the difference between the index RCG record and the previous RCG record was higher/lower than 10 mmol/l (representing extreme values greater than the 0.6^th^ centile of the RCG differences observed, suggesting potential measurement or recording error). To prepare the dataset for GBTM, we set four time intervals for sampling (see “Exposure” below and Additional file 1, Fig S2) and excluded those with RCG missing in more than one interval (*n* = 7047, or 8.5% of the otherwise eligible population). Ethics approval was obtained from the Stockholm regional ethical review committee (DNR 2010/1185–31/5, 2016/987–32). Informed consent was not required for the analysis of anonymized register data.

### Exposure

The recommended monitoring schedules for antenatal clinics in Stockholm for the relevant time period suggested monitoring RCG levels at five time points over the course of pregnancy, including the first visit (i.e., 10–12, 25, 29, 33, and 37 wkGA) [35]. We aimed to capture RCG values within each of four defined time intervals: ≤ 20, > 20–28, > 28–34, and > 34wkGA. The rationale was the following: (1) ≤ 20 wkGA captures glucose levels in early pregnancy, during which higher glucose may represent hyperglycemia first detected during pregnancy. (2) Physiologically, insulin resistance significantly increases around 24 wkGA in pregnancy, which may lead to hyperglycemia among those with insufficient insulin secretory capacity to maintain euglycemia [36]. We provided a buffer time of 4 weeks before and after this week to capture the measurements surrounding this time point which results in a category of > 20–28 wkGA. (3) We divided the third trimester at 34 wkGA which resulted in two groups, > 28–34 (early third trimester), and > 34 wkGA (late third trimester). Detailed information on RCG measurements during the entire pregnancy in our study was presented in Additional file 1, Table S1.

Because RCG is strongly correlated with the time interval between food intake and testing, we observed an overall pattern of RCG levels at different testing hours with a peak at 8:00 and a nadir at 12:00, and a peak at 13:00 and a nadir at 17:00 (Additional file 1, Fig S2). Therefore, we standardized the RCG levels into *z*-scores according to testing hour (Additional file 1, Fig S3), with the original RCG values in relation to the standardized *z*-scores shown in Additional file 1, Table S2. If women had RCG measured at multiple time points within an interval, we calculated the average RCG *z*-score for each gestational interval. The median frequency of measurements during each time interval was 1 (interquartile range [IQR]:1–1), 1 (1–2), 2 (1–2), and 1 (1–2), respectively, in line with the recommended monitoring schedules [35].

### Obstetric and neonatal outcomes

Obstetric and neonatal outcomes (Additional file 1, Table S3) were ascertained from (1) the Obstetrix system: gestational week-standardized total gestational weight gain *z*-scores (ZGWG), according to the Swedish standard [32], rate of gestational weight gain in the second trimester (RGWG-T2), in the third trimester (RGWG-T3); (2) the National Patient Register and MBR using International Classification of Diseases-10th revision (ICD-10): gestational hypertensive diseases, long labor time, obstructed labor, neonatal birth trauma, and neonatal hypoglycemia; (3) the MBR only: preterm birth (< 37wkGA), size for gestational age (i.e., small for gestational age [SGA], large for gestational age [LGA]), macrosomia (birthweight > 4500 g), mode of delivery (unassisted vaginal delivery, induced vaginal delivery, assisted vaginal delivery, and cesarean section), Apgar-score < 7 at 5-min; and (4) the Prescription Drug Register using Anatomical Therapeutic Chemical codes: antidiabetic treatments (i.e., glibenclamide/glyburide; metformin; insulin).

### Children’s neurodevelopmental conditions

Diagnoses of autism, ID, and ADHD were ascertained in SYC from all potential pathways to care in Stockholm County [33, 37, 38] (Additional file 1, Table S3). Services for children with NDCs are provided by services run by, or contracted by, Stockholm County and are available free of charge. Developmental surveillance is performed by specially trained child healthcare center nurses at regular intervals. Diagnostic evaluations are made by professional teams, typically consisting of at least a psychologist and a medical doctor at child pediatric or mental health services. ICD-10 diagnostic codes were used to identify the diagnoses: F84 for autism, F70-F79 for ID, and F90 for ADHD. The Prescription Drug Register was used to identify additional cases of ADHD when the child was prescribed with Methylphenidate or atomoxetine [38]. Our primary outcomes were “any autism”, “any ID”, “any ADHD”, and “any NDCs” when the child had any one of the diagnoses. As NDCs often co-occur with each other [6, 18], we generated three mutually exclusive outcomes, namely “only autism”, “only ADHD”, and “autism with ADHD” (Additional file 1, Fig S4). We did not have sufficient power to examine mutually exclusive diagnoses that included ID, due to the proportionally lower number of ID diagnoses compared to autism and ADHD.

### Covariates

The selection of potential confounders was based on previous studies that identified factors which have been associated with both exposure and outcomes, including the child’s sex [39], birth year [6], maternal age [1, 6], maternal education level [6, 40], maternal BMI measured in early pregnancy [1, 6], maternal birth region [40, 41], maternal psychiatric care before the birth of the index child [6], and parity [1] (Additional file 1, Table S3). Information on the earliest timing of OGTT, receipt of anti-diabetic treatment, and the diagnosis of GDM is also presented in Table 1, though not considered in the analysis as a confounder.

**Table 1:** Characteristics of the trajectory groups (*N* = 76,228)

	**Total population**	**Persistently low**	**Moderate**	**High in early pregnancy**	**High in mid-pregnancy**	**Persistently high**	***P*-value**^**a**^
**Total**		53,164	17,319	3178	1461	1106	
**Maternal characteristics**							
** Maternal age, mean (SD)**	31.3 (5.1)	31.3 (5.0)	31.5 (5.1)	31.9 (5.1)	31.5 (5.2)	31.8 (5.5)	< 0.001
** Maternal BMI, *n* (%)**							
Normal weight (18.5–24.9 kg/m^2^)	50,369 (66.1%)	36,532 (68.7%)	10,515 (60.7%)	2007 (63.2%)	856 (58.6%)	459 (41.5%)	< 0.001
Underweight (< 18.5 kg/m^2^)	2215 (2.9%)	1619 (3.0%)	424 (2.4%)	108 (3.4%)	36 (2.5%)	28 (2.5%)	
Overweight (25.0–29.9 kg/m^2^)	16,072 (21.1%)	10,456 (19.7%)	4209 (24.3%)	690 (21.7%)	349 (23.9%)	368 (33.3%)	
Obese (≥ 30 kg/m^2^)	6158 (8.1%)	3611 (6.8%)	1810 (10.5%)	316 (9.9%)	185 (12.7%)	236 (21.3%)	
*Missing*	1414 (1.9%)	946 (1.8%)	361 (2.1%)	57 (1.8%)	35 (2.4%)	15 (1.4%)	
** Maternal birth region, *n* (%)**							
Nordic	56,517 (74.1%)	41,104 (77.3%)	11,827 (68.3%)	2171 (68.3%)	885 (60.6%)	530 (47.9%)	< 0.001
Europe	4567 (6.0%)	3159 (5.9%)	1074 (6.2%)	188 (5.9%)	92 (6.3%)	54 (4.9%)	
Africa	3712 (4.9%)	1979 (3.7%)	1188 (6.9%)	232 (7.3%)	145 (9.9%)	168 (15.2%)	
Asia	9331 (12.2%)	5511 (10.4%)	2731 (15.8%)	501 (15.8%)	286 (19.6%)	302 (27.3%)	
Other	2094 (2.7%)	1405 (2.6%)	499 (2.9%)	86 (2.7%)	52 (3.6%)	52 (4.7%)	
*Missing*	7 (< 1%)	6 (0.01%)	0 (0.0%)	0 (0.0%)	< 5 (0.1%)	0 (0.0%)	
** Maternal psychiatric history, *n* (%)**	7898 (10.4%)	5460 (10.3%)	1834 (10.6%)	315 (9.9%)	156 (10.7%)	133 (12.0%)	0.23
** Maternal education level, *n* (%)**							
Pre-high school	8066 (10.6%)	5220 (9.8%)	2066 (11.9%)	351 (11.0%)	222 (15.2%)	207 (18.7%)	< 0.001
High school	24,484 (32.1%)	16,743 (31.5%)	5748 (33.2%)	1088 (34.2%)	489 (33.5%)	416 (37.6%)	
Post-high school	43,232 (56.7%)	30,896 (58.1%)	9398 (54.3%)	1720 (54.1%)	742 (50.8%)	476 (43.0%)	
*Missing*	446 (0.6%)	305 (0.6%)	107 (0.6%)	19 (0.6%)	8 (0.5%)	7 (0.6%)	
** Parity, *n* (%)**							
Nulliparous	35,490 (46.6%)	25,015 (47.1%)	8079 (46.6%)	1240 (39.0%)	667 (45.7%)	489 (44.2%)	< 0.001
1	27,995 (36.7%)	19,665 (37.0%)	6224 (35.9%)	1264 (39.8%)	489 (33.5%)	353 (31.9%)	
≥ 2	12,743 (16.7%)	8484 (16.0%)	3016 (17.4%)	674 (21.2%)	305 (20.9%)	264 (23.9%)	
** GDM, *n* (%)**	219 (0.3%)	15 (0.03%)	42 (0.2%)	24 (0.8%)	36 (2.5%)	102 (9.2%)	< 0.001
** RGWG-T2, mean (SD)**	0.58 (0.27)	0.57 (0.27)	0.58 (0.29)	0.56 (0.29)	0.58 (0.30)	0.58 (0.33)	< 0.001
** RGWG-T3, mean (SD)**	0.52 (0.24)	0.52 (0.24)	0.54 (0.24)	0.50 (0.24)	0.52 (0.25)	0.55 (0.29)	< 0.001
** Total GWG z-scores, mean (SD)**	0.09 (1.02)	0.07 (1.02)	0.16 (1.03)	− 0.00 (1.02)	0.10 (1.09)	0.22 (1.18)	< 0.001
** Antidiabetic treatment, *n* (%)**							
No treatment	76,094 (99.8%)	53,132 (99.9%)	17,294 (99.9%)	3162 (99.5%)	1451 (99.3%)	1055 (95.4%)	< 0.001
≤ 20 wkGA	53 (0.1%)	30 (0.1%)	8 (0.05%)	10 (0.3%)	0 (0.0%)	5 (0.5%)	
> 20–28 wkGA	6 (< 1%)	< 5 (0.002%)	0 (0.0%)	< 5 (0.1%)	< 5 (0.1%)	< 5 (0.2%)	
> 28–34 wkGA	36 (< 1%)	0 (0.0%)	5 (0.03%)	< 5 (0.1%)	6 (0.4%)	23 (2.1%)	
> 34 wkGA	39 (0.1%)	< 5 (0.002%)	12 (0.1%)	< 5 (0.1%)	< 5 (0.2%)	21 (1.9%)	
** The earliest OGTT, *n* (%)**							
No OGTT	74,104 (97.2%)	52,371 (98.5%)	16,667 (96.2%)	2954 (93.0%)	1276 (87.3%)	836 (75.6%)	< 0.001
≤ 20 wkGA	289 (0.4%)	81 (0.2%)	49 (0.3%)	129 (4.1%)	8 (0.5%)	22 (2.0%)	
> 20–28 wkGA	513 (0.7%)	172 (0.3%)	132 (0.8%)	36 (1.1%)	125 (8.6%)	48 (4.3%)	
> 28–34 wkGA	971 (1.3%)	416 (0.8%)	317 (1.8%)	44 (1.4%)	48 (3.3%)	146 (13.2%)	
> 34 wkGA	351 (0.5%)	124 (0.2%)	154 (0.9%)	15 (0.5%)	< 5 (0.3%)	54 (4.9%)	
** Gestational hypertensive diseases, *n* (%)**	3742 (4.9%)	2525 (4.7%)	853 (4.9%)	157 (4.9%)	106 (7.3%)	101 (9.1%)	< 0.001
** Mode of delivery, *n* (%)**							
Unassisted vaginal delivery	47,417 (62.2%)	33,727 (63.4%)	10,340 (59.7%)	1954 (61.5%)	838 (57.4%)	558 (50.5%)	< 0.001
Induced vaginal delivery	7046 (9.2%)	4855 (9.1%)	1595 (9.2%)	315 (9.9%)	148 (10.1%)	133 (12.0%)	
Assisted vaginal delivery	7061 (9.3%)	4894 (9.2%)	1646 (9.5%)	279 (8.8%)	137 (9.4%)	105 (9.5%)	
Elective cesarean section	7664 (10.1%)	5141 (9.7%)	1879 (10.8%)	333 (10.5%)	169 (11.6%)	142 (12.8%)	
Emergency cesarean section	7040 (9.2%)	4547 (8.6%)	1859 (10.7%)	297 (9.3%)	169 (11.6%)	168 (15.2%)	
** Long labor time, *n* (%)**	1746 (2.3%)	1156 (2.2%)	449 (2.6%)	66 (2.1%)	39 (2.7%)	36 (3.3%)	0.002
** Obstructed labor,* n* (%)**	704 (0.9%)	416 (0.8%)	208 (1.2%)	36 (1.1%)	19 (1.3%)	25 (2.3%)	< 0.001
**Children’s characteristics**							
** Child’s sex, *n* (%)**							
Male	39,281 (51.5%)	27,328 (51.4%)	8978 (51.8%)	1625 (51.1%)	734 (50.2%)	616 (55.7%)	0.043
Female	36,947 (48.5%)	25,836 (48.6%)	8341 (48.2%)	1553 (48.9%)	727 (49.8%)	490 (44.3%)	
** Gestational week at birth, *n* (%)**							
Preterm (< 37 weeks)	2544 (3.3%)	1759 (3.3%)	531 (3.1%)	117 (3.7%)	69 (4.7%)	68 (6.1%)	< 0.001
Term (37– < 42 weeks)	68,870 (90.3%)	48,062 (90.4%)	15,677 (90.5%)	2853 (89.8%)	1305 (89.3%)	973 (88.0%)	
Post-term (≥ 42 weeks)	4814 (6.3%)	3343 (6.3%)	1111 (6.4%)	208 (6.5%)	87 (6.0%)	65 (5.9%)	
** Neonatal birth trauma, *n* (%)**	1322 (1.7%)	817 (1.5%)	380 (2.2%)	66 (2.1%)	28 (1.9%)	31 (2.8%)	< 0.001
** Size for gestational age, *n* (%)**							
AGA	72,382 (95.0%)	50,740 (95.4%)	16,312 (94.2%)	3000 (94.4%)	1350 (92.4%)	980 (88.6%)	< 0.001
SGA	1493 (2.0%)	1085 (2.0%)	287 (1.7%)	73 (2.3%)	32 (2.2%)	16 (1.4%)	
LGA	2223 (2.9%)	1254 (2.4%)	684 (3.9%)	98 (3.1%)	78 (5.3%)	109 (9.9%)	
*Missing*	130 (0.2%)	85 (0.2%)	36 (0.2%)	7 (0.2%)	< 5 (0.1%)	< 5 (0.1%)	
** Macrosomia (birthweight > 4500 g), *n* (%)**							
Yes	2328 (3.1%)	1380 (2.6%)	701 (4.0%)	108 (3.4%)	61 (4.2%)	78 (7.1%)	< 0.001
* Missing*	130 (0.2%)	85 (0.2%)	36 (0.2%)	7 (0.2%)	< 5 (0.1%)	< 5 (0.1%)	
**Apgar score at 5-min < 7, *n* (%)**							
Yes	503 (0.7%)	327 (0.6%)	134 (0.8%)	20 (0.6%)	11 (0.8%)	11 (1.0%)	0.10
*Missing*	241 (0.3%)	160 (0.3%)	62 (0.4%)	15 (0.5%)	< 5 (0.2%)	< 5 (0.1%)	
** Neonatal hypoglycemia, *n* (%)**	1637 (2.1%)	993 (1.9%)	433 (2.5%)	101 (3.2%)	39 (2.7%)	71 (6.4%)	< 0.001
** Children’s NDCs, *n* (%)**							
Any NDCs	2890 (3.8%)	1929 (3.6%)	709 (4.1%)	132 (4.2%)	65 (4.4%)	55 (5.0%)	0.004
Any autism	1465 (1.9%)	997 (1.9%)	352 (2.0%)	66 (2.1%)	30 (2.1%)	20 (1.8%)	0.64
Any ID	404 (0.5%)	260 (0.5%)	104 (0.6%)	26 (0.8%)	9 (0.6%)	5 (0.5%)	0.066
Any ADHD	1758 (2.3%)	1160 (2.2%)	436 (2.5%)	84 (2.6%)	38 (2.6%)	40 (3.6%)	0.001
Autism only	820 (1.1%)	571 (1.1%)	188 (1.1%)	30 (0.9%)	19 (1.3%)	12 (1.1%)	0.87
ADHD only	1238 (1.6%)	809 (1.5%)	318 (1.8%)	51 (1.6%)	29 (2.0%)	31 (2.8%)	< 0.001
Autism and ADHD	428 (0.6%)	289 (0.5%)	99 (0.6%)	25 (0.8%)	8 (0.5%)	7 (0.6%)	0.49

*Abbreviations: GDM* gestational diabetes mellitus, *RGWG-T2* rate of gestational weight gain in the second trimester (g/week), *RGWG-T3* rate of gestational weight gain in the third trimester (g/week), *Total GWG z-score* total gestational weight gain (standardized by the gestational duration), *ID* intellectual disability, *ADHD* attention-deficit hyperactivity disorder, *NDC* neurodevelopmental conditions, *AGA* appropriate for gestational age, *SGA* small for gestational age, *LGA* large for gestational age, *wkGA* weeks of gestational age, *SD* standard deviation, *IQR* interquartile range

^a^We applied χ^2^ tests for proportions and ANOVA tests for means to compare NDC diagnoses and maternal, obstetric, neonatal, and children’s characteristics among different trajectory groups

### Statistical analysis

Stata 16.0 was used for data analysis. Group-based trajectory modeling (GBTM) was performed using the Stata Plugin “traj” with a censored normal modeling. Average RCG *z*-scores for the four defined time intervals were used to identify individuals following approximately the same pattern over time. GBTM is based on the assumption that the population is composed of distinct groups or patterns that can be identified to show a different underlying trajectory over time. It further assumes that there is no variation between individuals within the same group [42]. Maximum likelihood was used for the estimation of the model parameters. Individuals with some missing longitudinal data values are included in the analysis using full information maximum likelihood (FIML) [43]. Models capture both trajectory shape and individual membership in relation to different groups. We balanced information from model fit indices (e.g., Bayesian information criteria [BIC] and entropy) with previously defined criteria for model adequacy [43]. We first assessed the best-fitting model using BIC after fitting different permutations of polynomials (from linear, quadratic to cubic, and from 1 to 6 groups). Though a higher BIC value (less negative) was preferred, we also required the following [43]: (1) reasonable sample size for each group (over 1000 observations); (2) narrow confidence intervals in post-estimation plotting; (3) close correspondence between the estimated probability of group membership and the proportion assigned to that group based on the posterior probability of group membership; (4) the average of the posterior probabilities of group membership exceeds a minimum threshold of 0.7; (5) the odds of correct classification based on the posterior probabilities of group membership exceed 5. Detailed information on model selection was described in Additional file 1, Supplemental Methods [44–46], Tables S4-S5, Figures S5-S6. To achieve global maxima, after we ran the final selected model, we generated a new matrix of random start values using 0.002 as the amount of variability and reran the model with 10,000 iterations. We reported the results of GBTM according to the GRoLTS-Checklist for reporting on latent trajectory studies [47] (Additional file 2). After conducting the GBTM, we used “trajplot” in Stata to plot the trajectory groups. We then randomly selected 110 observations from each group and created a spaghetti plot with exact RCG z-scores.

The characteristics of the five trajectory groups were compared using the *χ*^2^ tests for proportions, ANOVA tests for means, and Kruskal–Wallis tests for medians. No adjustments were made for the purpose of description. Statistical significance was defined as *P* < 0.05.

The odds ratios (ORs) and 95% confidence intervals (CIs) were calculated for the associations between different trajectory groups and both obstetric/neonatal complications and children’s NDC outcomes using generalized estimating equation (GEE) models with logit link clustered on maternal identification number. We replaced the missing values in confounders with a dummy category. For model 1, we adjusted for birth year and child’s sex. For model 2, we further adjusted for maternal age, education level, BMI, birth region, and parity, with maternal psychiatric history included in model 2 only when analyzing associations with NDCs.

In both Model 1 and Model 2 for obstetric/neonatal and NDC outcomes, we employed the Benjamini-Hochberg (BH) procedure [48] for controlling the false discovery rate (FDR) which entailed the correction of p-values in the context of multiple comparisons, thereby facilitating efficient control of the FDR.

### Sensitivity analysis

The Stata “traj” plugin uses FIML to handle missing values. To investigate the influence of missing values during different time periods on the model’s parameter estimation and adequacy, we dropped observations with missing values or replaced them with extreme values (the smallest and largest values) during certain periods and replicated the analysis. To disentangle the effect of overweight/obesity in the associations with different outcomes, we further excluded those with overweight/obesity and replicated our analyses. Furthermore, to investigate if the associations with offspring NDCs could be influenced by gestational hypertensive diseases, we excluded those exposed to gestational hypertensive diseases and replicated our analyses.

## Results

### Population characteristics

Our final study sample included 76,228 children born to 68,768 mothers. GDM was diagnosed in 219 (0.3%) of the observed pregnancies. At the end of follow-up, the median age of the children was 7.6 (IQR 6.7–8.4) years old, among whom 1465 (1.96%) were diagnosed with autism, 1758 (2.34%) with ADHD, and 404 (0.55%) with ID (Table 1). Compared to those excluded because of missing RCG values, those included in the final sample had lower proportions of NDCs and overweight/obese mothers, and higher proportions of well-educated and Nordic-born mothers (Additional file 1, Table S6). Of those included, 5.84%, 3.93%, 5.24%, and 9.44% were missing maternal RCG values within the time intervals of ≤ 20, > 20–28, > 28–34, and > 34 wkGA, respectively. 71.5% children had complete records for maternal RCG across the four time intervals. Those missing RCG during each time interval differed from with observations, with patterns specific to each time interval (Additional file 1, Table S7). For example, children missing > 34 wkGA measurements were more likely to be born preterm compared to those with measurements (26.4% vs. 0.9%, *P* < 0.001), and children with missing values in the ≤ 20 and > 34 wkGA intervals were more likely to be diagnosed with an NDC compared to those without missing values (4.7% vs. 3.7% [*P* = 0.002] and 4.5% vs. 3.7% [*P* < 0.001], respectively). Children who were affected by NDCs differed from those unaffected in all covariates, except macrosomia, patterns of OGTT screening, and neonatal birth trauma (Additional file 1, Table S8).

### Trajectory model selection

**Fig. 1:**
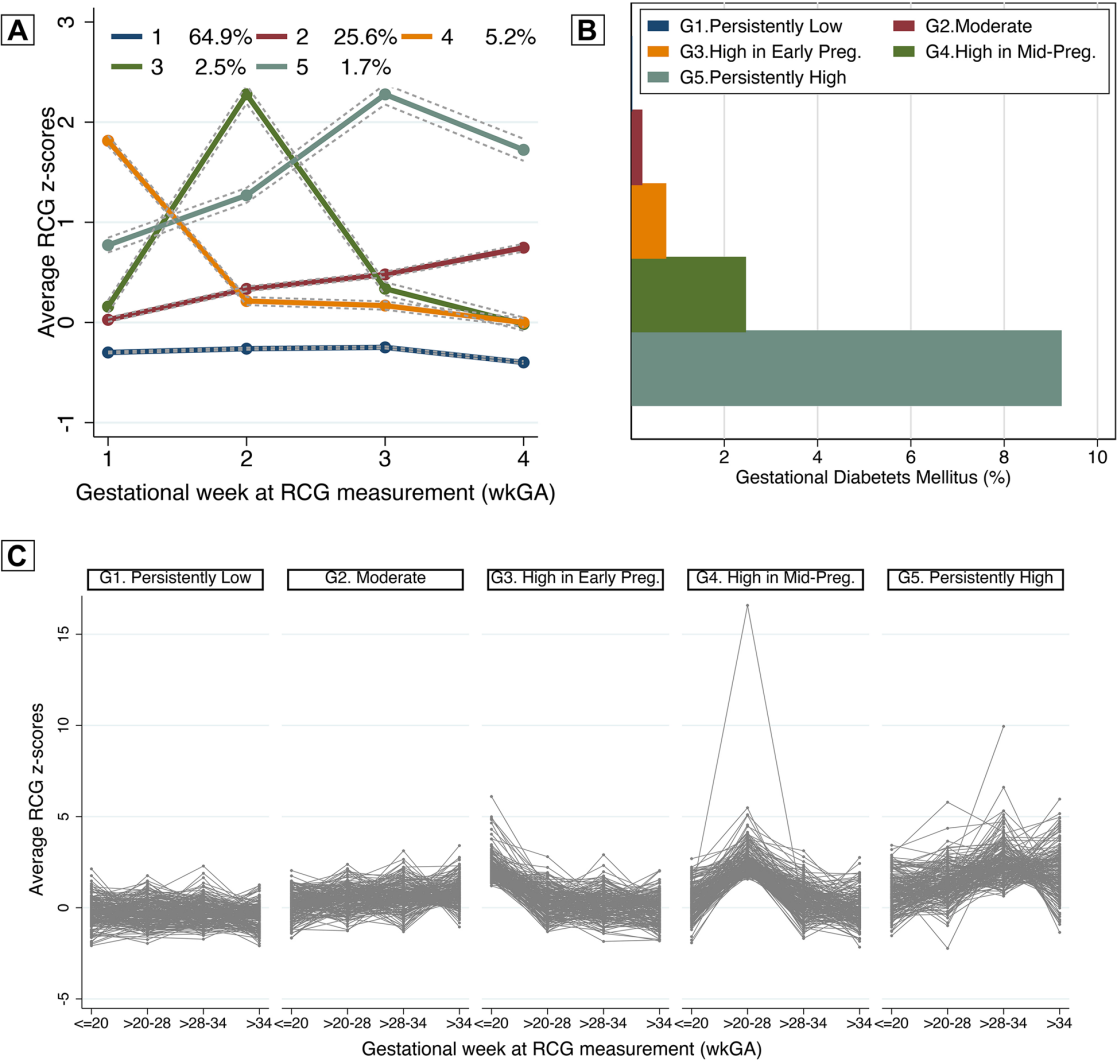
RCG *z*-score trajectory groups throughout pregnancy. **A** RCG *z*-score trajectory groups throughout pregnancy. **B** Proportions of Gestational diabetes mellitus in each trajectory group **C**  To visualize the accuracy of assignment, Lasagne plots depicting the observed ZRCG throughout pregnancy for 110 randomly selected pregnancies from each trajectory group (restricted to aid the clarity of the plot) were compared to post-estimation plots

Our final model ultimately consisted of 5 groups with 5 cubic trajectories, as shown in Fig. 1A. Additional information on the parameter estimations, variance–covariance matrix, matrix of misclassification errors and model adequacy can be found in Additional file 1, Supplementary Methods, Table S9-12. Our final model had a reasonable sample size for each group (over 1000 observations) and narrow confidence intervals in post-estimation plotting (Fig. 1A) with good correspondence between trajectory groups and observed patterns of glucose measurements (Fig. 1C). We observed close correspondence between the estimated probability of group membership and the proportion assigned to that group based on the posterior probability of group membership. The average of the posterior probabilities of group membership exceeded a minimum threshold of 0.7, and the odds of correct classification based on the posterior probabilities of group membership exceeded 5 for most of the groups, except for Group 1 (“Persistently Low”).

### Patterns of distinct glucose trajectory groups

Group 1 (referent group, “Persistently Low”) included 53,164 observations (64.9% of the cohort) with relatively low and stable maternal RCG levels throughout pregnancy. Group 2 (“Moderate”) included 17,319 observations (25.6%) with a slightly higher maternal RCG at the baseline compared to Group 1 that slowly increased throughout pregnancy without an obvious peak, indicating a moderately higher RCG level, more likely to represent a subclinical hyperglycemic state. Group 3 (“High in Early Pregnancy”) included 3178 observations (5.2%) with a high maternal RCG at baseline followed by a steep decrease from 20 wkGA until the end of pregnancy. Group 4 (“High in Mid-Pregnancy”) included 1461 observations (2.5%) with a slightly higher baseline maternal RCG compared to Group 1, followed by a sharp increase between 20 and 28 wkGA with a return to baseline level afterwards. Group 5 (“Persistently High”) included 1106 observations (1.7%) with a relatively high maternal RCG at the baseline, reaching a peak in the early third trimester followed by a slight drop at the end of pregnancy.

### Characteristics of the distinct glucose trajectory groups

The proportions of maternal obesity varied in the trajectory groups, with 10.5%, 9.9%, 12.7%, and 21.3% in Group 2 (“Moderate”), Group 3 (“High in Early Pregnancy”), Group 4 (“High in Mid-Pregnancy”), and Group 5 (“Persistently High”), respectively (*p* < 0.001; Table 1). The proportion of Nordic-born mothers was 77.3% in Group 1, decreasing to 68.3%, 68.3%, 60.6%, and 47.9% in Groups 2 to 5, respectively (*p* < 0.001). Similarly, the proportion of women with pre-high school education in Group 1 was 9.8%, increasing to 11.9%, 11.0%, 15.2%, and 18.7% in Groups 2 to 5, respectively (*p* < 0.001). The rates of weight gain in trimesters 2 and 3 differed across different trajectory groups (*p* < 0.001 for both trimesters), with the lowest rate of weight gain in both trimesters and the lowest total weight gain z-scores observed in Group 3 (“High in Early Pregnancy”). In Group 1, the proportion of GDM was 0.03%, while the proportions were 0.2%, 0.8%, 2.5%, and 9.2% in Groups 2 to 5, respectively. In Group 1, the proportion of OGTT referral was 1.5%, while the proportions were 3.8%, 7.0%, 12.7%, and 24.4% in Groups 2 to 5, respectively (Table 1). The proportion of individuals receiving antidiabetic treatments was generally low across different trajectory groups, though the proportion of those receiving treatment and the timing of the treatment did seem to vary across the groups. In Group 1, the proportion of antidiabetic treatment was 0.1%, which was similar to the proportion in Group 2. However, the proportions increased to 0.5%, 0.7%, and 4.6% in Groups 3 to 5, respectively. Women in Group 3 (“High in Early Pregnancy”), which had a drop in glucose level after hyperglycemia identified ≤ 20 wkGA, were more likely to be treated in early pregnancy (≤ 20 wkGA). Women in Group 4 (“High in Mid-Pregnancy”), which had a drop in glucose level after hyperglycemia identified > 20–28 wkGA, were more likely to receive antidiabetic treatments during the early third trimester. Finally, women in Group 5, which had a peak in early third trimester between > 28–34 wkGA followed by a slight drop in RCG level, were more likely to receive treatment after 28 wkGA.

### Maternal glucose trajectory groups and obstetric and neonatal outcomes

#### Maternal outcomes

Compared to Group 1 (“Persistently Low”), Group 2 (“Moderate”) was not associated with gestational hypertensive diseases, or induced vaginal delivery, but was associated with assisted vaginal delivery (OR 1.08, 95% CI 1.02–1.15, *P* = 0.013), cesarean section (OR 1.15, 95% CI 1.10–1.20, *P* < 0.001), long labor time (OR 1.14, 95% CI 1.02–1.28, *P* = 0.023), and obstructed labor (OR 1.45, 95% CI 1.22–1.71, *P* < 0.001). However, all these associations remained significant after accounting for FDR except for the long labor time.

Group 3 (“High in Early Pregnancy”) was not associated with gestational hypertensive diseases, induced vaginal delivery, assisted vaginal delivery, or long labor time, but was associated with cesarean section (OR 1.11, 95% CI 1.01–1.21, *P* = 0.029), and obstructed labor (OR 1.51, 95% CI 1.07–2.13, *P* = 0.018). However, neither of the associations remained significant after accounting for FDR.

Group 4 (“High in Mid-pregnancy”) was not associated with induced vaginal delivery, assisted vaginal delivery, long labor time, or obstructed labor, but was associated with gestational hypertensive diseases (OR 1.48, 95% CI 1.20–1.82, *P* < 0.001), and cesarean section (OR 1.24, 95% CI 1.09–1.40, *P* = 0.001), which remained significant after accounting for FDR.

**Fig. 2:**
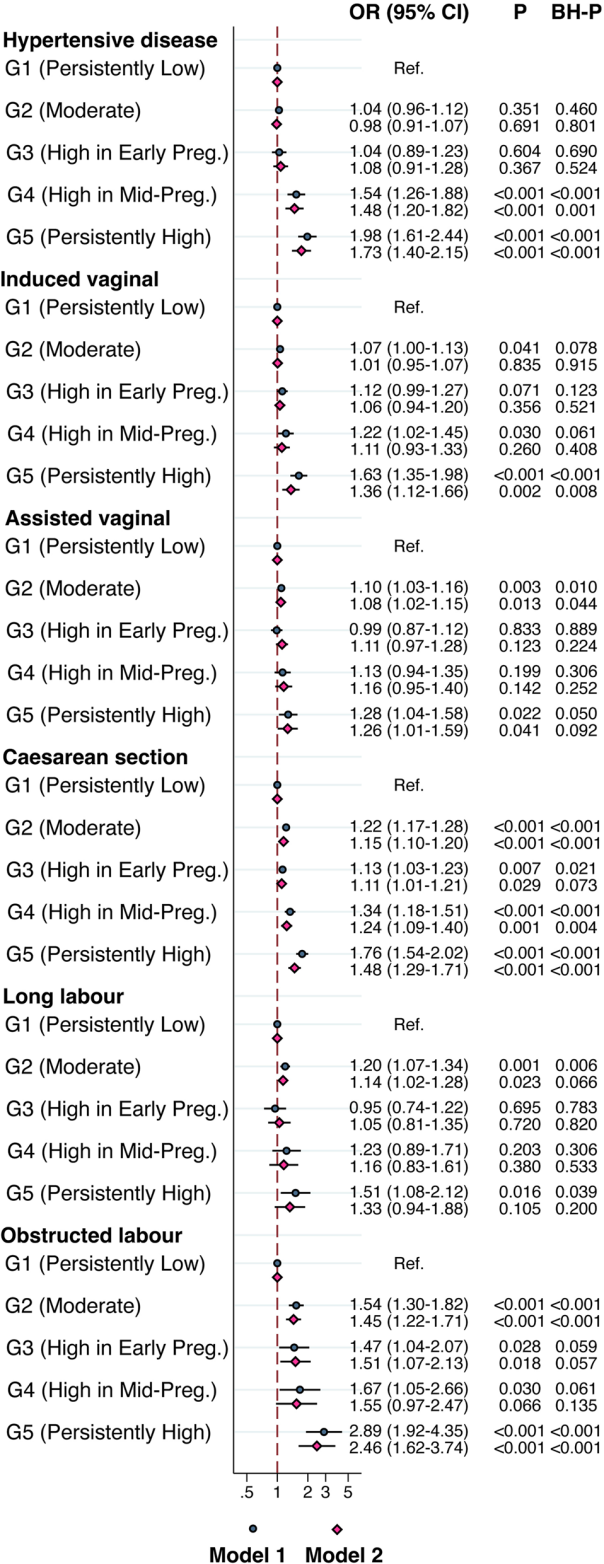
Odds ratios (ORs) and 95% confidence intervals (CIs) for the associations between maternal RCG *z*-score trajectory groups and maternal outcomes. GEE model, clustered on maternal identification numbers. Model 1: adjusted for child’s sex, birth year. Model 2: adjusted for model 1 and maternal birth region, maternal age, maternal education level, maternal BMI, and parity. *P*-values after applying the Benjamini-Hochberg (BH) procedure for controlling FDR are presented

Group 5 (“Persistently High”) was not associated with long labor time but was associated with all the other maternal outcomes. However, only associations with gestational hypertensive diseases (OR 1.73, 95% CI 1.40–2.15, *P* < 0.001), induced vaginal delivery (OR 1.36, 95% CI 1.12–1.66, *P* = 0.002), cesarean section (OR 1.48, 95% CI 1.29–1.71, *P* < 0.001), and obstructed labor (OR 2.46, 95% CI 1.62–3.74, *P* < 0.001) remained significant after accounting for FDR (Fig. 2).

#### Neonatal outcomes

Compared to Group 1 (“Persistently Low”), Group 2 (“Moderate”) was not associated with increased risk of 5-min Apgar score < 7, but associated with all the other neonatal outcomes. However, only the associations with SGA (OR 0.74, 95% CI 0.65–0.85, *P* < 0.001), LGA (OR 1.61, 95% CI 1.46–1.77, *P* < 0.001), macrosomia (OR 1.55, 95% CI 1.41–1.71, *P* < 0.001), neonatal birth trauma (OR 1.40, 95% CI 1.23–1.58, *P* < 0.001), and neonatal hypoglycemia (OR 1.23, 95% CI 1.09–1.38, *P* = 0.001) remained significant after accounting for FDR.

Group 3 (“High in Early Pregnancy”) was not associated with preterm birth, SGA, or 5-min Apgar score < 7, but associated with LGA, macrosomia, neonatal birth trauma, and neonatal hypoglycemia. However, only the association with macrosomia (OR 1.29, 95% CI 1.05–1.57, *P* = 0.014), neonatal birth trauma (OR 1.42, 95% CI 1.10–1.83, *P* = 0.007), and neonatal hypoglycemia (OR 1.66, 95% CI 1.35–2.05, *P* < 0.001) remained significant after accounting for FDR.

Group 4 (“High in Mid-Pregnancy”) was not associated with SGA, neonatal birth trauma, 5-min Apgar score < 7, or neonatal hypoglycemia, but associated with preterm birth (OR 1.38, 95% CI 1.08–1.77, *P* = 0.011), LGA (OR 2.28, 95% CI 1.79–2.89, *P* < 0.001), and macrosomia (OR 1.65, 95% CI 1.26–2.15, *P* < 0.001). All these associations remained significant after accounting for FDR.

**Fig. 3:**
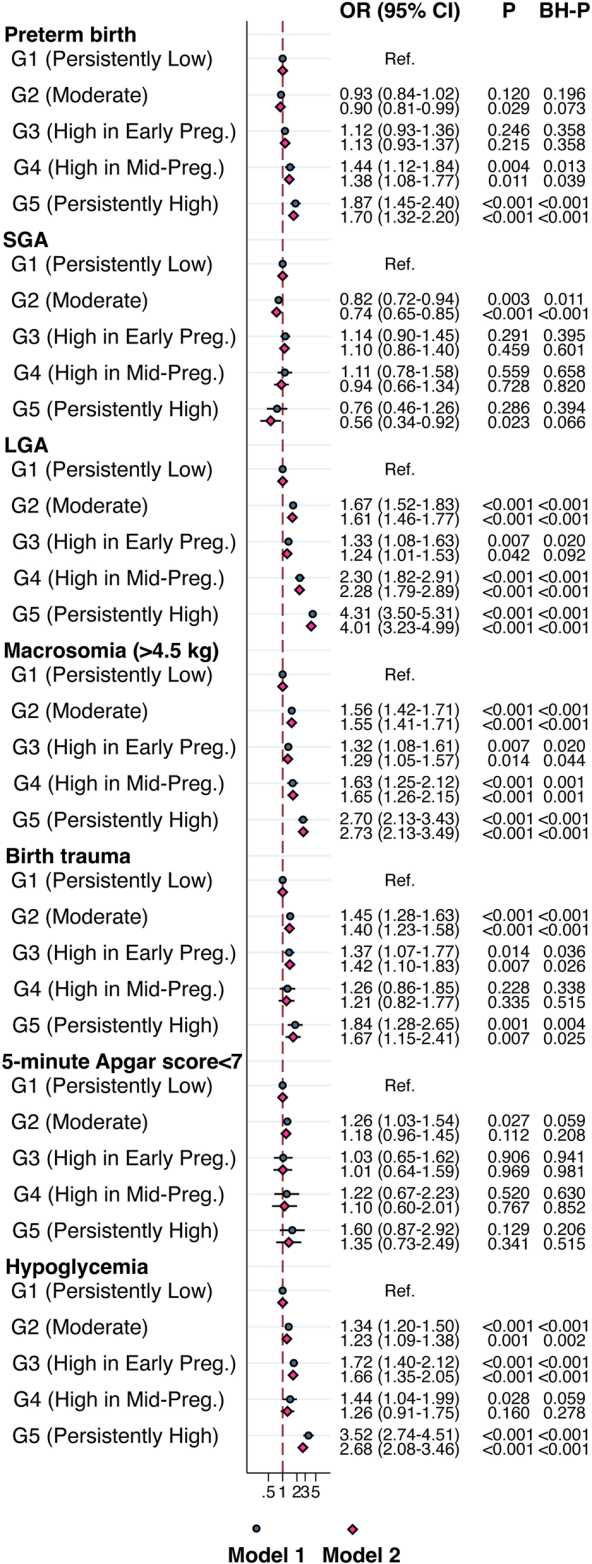
Odds ratios (ORs) and 95% confidence intervals (CIs) for the associations between maternal RCG *z*-score trajectory groups and neonatal complications. GEE model, clustered on maternal identification numbers. Model 1: adjusted for child’s sex, birth year. Model 2: adjusted for model 1 and maternal birth region, maternal age, maternal education level, maternal BMI, and parity. *P*-values after applying the Benjamini-Hochberg (BH) procedure for controlling FDR are presented

Group 5 (“Persistently High”) was not associated with 5-min Apgar score < 7 but associated with all the other neonatal outcomes, including preterm birth (OR 1.70, 95% CI 1.32–2.20, *P* < 0.001), SGA (OR 0.56, 95% CI 0.34–0.92, *P* = 0.023), LGA (OR 4.01, 95% CI 3.23–4.99, *P* < 0.001), macrosomia (OR 2.73, 95% CI 2.13–3.49, *P* < 0.001), neonatal birth trauma (OR 1.67, 95% CI 1.15–2.41,* P* = 0.007), and neonatal hypoglycemia (OR 2.68, 95% CI 2.08–3.46, *P* < 0.001). All these associations remained significant after accounting for FDR except for SGA (Fig. 3).

### Maternal glucose trajectory groups and children’s risks of NDCs

**Fig. 4:**
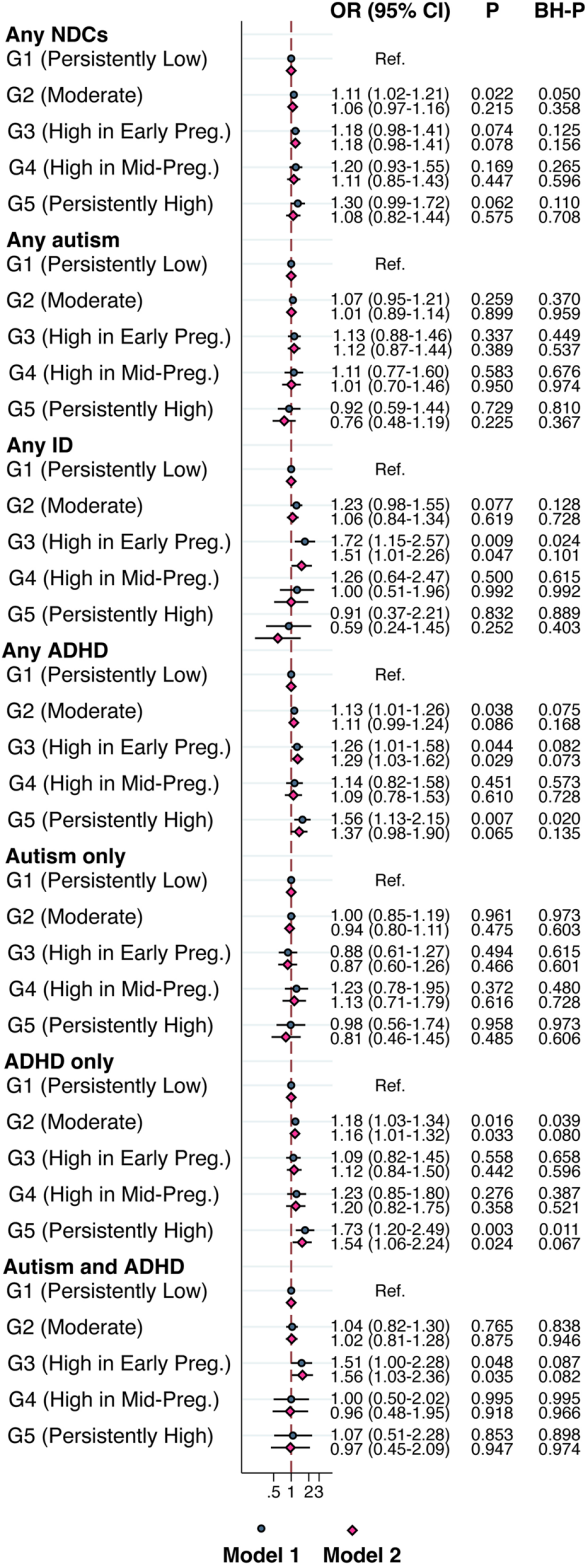
Odds ratios (ORs) and 95% confidence intervals (CIs) for the associations between maternal RCG *z*-score trajectory groups and NDCs in offspring. GEE model, clustered on maternal identification numbers. Model 1: adjusted for child’s sex and birth year. Model 2: adjusted for model 1 and maternal birth region, maternal age, maternal education level, maternal BMI, maternal psychiatric history, and parity. *P*-values after applying the Benjamini-Hochberg (BH) procedure for controlling FDR are presented

Compared to Group 1 (“Persistently Low”), Group 2 (“Moderate”) was associated with “ADHD only” (OR 1.16, 95% CI 1.01–1.32, *P* = 0.033), but not associated with any other NDCs. Group 3 (“High in Early Pregnancy”) was associated with “Any ID” (OR 1.51, 95% CI 1.01–2.26, *P* = 0.047), “Any ADHD” (OR 1.29, 95% CI 1.03–1.62, *P* = 0.029), and “Autism and ADHD” (OR 1.56, 95% CI 1.03–2.36, *P* = 0.035), but not associated with any other NDCs. Group 4 (“High in Mid-Pregnancy”) was not associated with any of these NDCs. Group 5 (“Persistently High”) was associated with “ADHD only” (OR 1.54, 95% CI 1.06–2.24,* P* = 0.024), but not associated with any other NDCs. However, none of the associations with NDC outcomes remained significant after accounting for FDR (Fig. 4).

### Sensitivity analysis

After excluding individuals with missing RCG values at any of the four intervals (*N*_excluded_ = 18,652), we observed the same pattern for the five trajectory groups (Additional file 1, Fig S7B), albeit with a better model adequacy (Additional file 1, Table S12). Replacing missing values with either the smallest or largest values of each time interval did not significantly change the shape or adequacy of trajectory models (Additional file 1, Fig S7C- S7D, and Table S12). After excluding individuals with overweight and obese mothers, associations with obstetric and neonatal outcomes remained broadly similar to the main findings, except for the associations with severe outcomes (e.g., gestational hypertensive disease, preterm birth) and induced vaginal delivery, which became insignificant after FDR adjustment (Additional file 1, Table S13). The associations between “Group 3” and both “Any ADHD” and “Autism and ADHD” became statistically significant (Additional file 1, Table S13). After excluding individuals exposed to gestational hypertensive diseases, the associations with offspring NDCs largely remained similar to the main findings (Additional file 1, Table S14).

## Discussion

We observed five distinct patterns of glucose trajectories throughout pregnancy. Higher odds of obstetric and neonatal complications were not only associated with the “Persistently High” group, but also with the “Moderate,” “High in Early Pregnancy,” and “High in Mid-Pregnancy” groups to differing degrees compared to the referent “Persistently Low” group. These trajectory groups, except for the “High in Mid-Pregnancy” group, were also associated with increased children’s odds of NDCs in the long term, though none of these associations remained statistically significant after accounting for FDR.

### Interpretation of findings

Previous research on maternal hyperglycemia and related risks primarily focused on clinical GDM diagnosis. Although some studies linked subclinical glucose levels to obstetric/neonatal complications, they relied on single-point measurements [5, 49, 50]. One study found associations between high fasting glucose trajectories and perinatal outcomes (i.e., LGA and macrosomia) [51]. However, no studies, to our knowledge, explored the association between glucose trajectories across pregnancy and children’s NDC risk.

Group 2, featuring subclinical hyperglycemia with glucose levels ranging from 0 to + 1 standard deviation (SD), and low GDM diagnosis rate, still indicated raised risks of fetal overgrowth and birth complications, albeit not severe complications (e.g., gestational hypertensive diseases and preterm birth), consistent with the associations observed for mid-pregnancy glucose levels reported in the HAPO study [5]. Even after excluding overweight/obese mothers, these associations remained, emphasizing that persistent subclinical hyperglycemia could influence fetal growth and birth even in a low-risk group. Given IADPSG’s GDM diagnostic cut-offs, this group might be overlooked in clinical settings, yet clinical trial reviews indicate dietary advice and glucose monitoring can mitigate macrosomic and LGA babies in this group without raising operation rates [52]. However, current GDM diagnostic criteria necessitate updated clinical trials [53]. Therefore, expectant mothers with moderately high glucose levels, typically undiagnosed with GDM, need clinical attention and potential lifestyle interventions.

Early pregnancy hyperglycemia (Group 3), marked by a glucose peak near + 2 SD, could be due to overt diabetes first identified in pregnancy [23]. The weight gain rates in this group, suggesting response to early pregnancy interventions, were not as high as in other groups. This group did not display increased risk for severe outcomes like gestational hypertensive diseases or preterm birth, indicating early glucose management benefits. Macrosomia and neonatal hypoglycemia risks persisted even after excluding overweight/obese mothers. Potential mechanisms might involve early onset fetal hyperinsulinemia influencing fetal glucose uptake, which could potentially mask a GDM diagnosis [54]. Mitigating fetal hyperinsulinemia effects in cases of early hyperglycemia may be challenging [54].

Mothers with high RCG levels peaking above + 2 SD in mid-pregnancy (Group 4) showed increased odds for several complications, though less than persistently high RCG group, underscoring potential benefits of glucose management after high mid-pregnancy levels detection. Previous studies showed that optimal GDM management can reduce perinatal complications like preeclampsia [30] and preterm birth [29]. Mid-pregnancy-focused screening programs could detect this group effectively as well as those with persistently high levels. Our results distinguished risk levels for persistently high glucose levels from transiently high mid-pregnancy levels, revealing higher risks in the former, adding nuance to the insights provided by studies which focus on mid-pregnancy [5]. Even after excluding overweight/obese mothers, LGA and macrosomia associations persisted, suggesting mid-pregnancy hyperglycemia can influence fetal growth, even with later glycemic control.

The “Persistently High” group (Group 5), with glucose levels on average initially at + 1 SD and escalating beyond + 2 SD in late pregnancy, showed the highest associations with obstetric and neonatal complications. These observations aligned with the known risks of slightly elevated glucose levels and overt GDM (though less than 10% of mothers in this group were diagnosed with GDM) [5]. These risks regarding fetal overgrowth and birth complications persisted even when mothers with overweight/obesity were excluded. Despite this, only 4.6% received antidiabetic treatments, mostly post-28 wkGA, which aligned with a minor drop in RCG levels. Therefore, for this group, earlier monitoring and intervention may be crucial.

None of the associations with offspring NDCs remained significant after FDR corrections given our small sample size in which to study rare NDC outcomes. Here we cautiously note several associations that are consistent with previous findings in the literature for overt GDM diagnoses that warrant further exploration in larger studies. Both Group 2 (0 to + 1 SD glucose levels) and Group 5 (+ 1 SD early, above + 2 SD late) showed “ADHD only” associations, more so in Group 5. These findings aligned with prior GDM studies and Xiang et al.’s increased ADHD risk with medicated GDM (considered as a marker of severe GDM), though the efficacy of treatment was unclear [13]. Group 4 had no NDC associations, implying mid-pregnancy glycemic control’s benefits to fetal neurodevelopment. However, we observed signals indicating that early pregnancy hyperglycemia was associated with NDCs despite subsequent glycemic control, including an association after excluding those with maternal overweight/obesity. Mechanisms to explain an association between maternal hyperglycemia and children’s risks of NDCs remain unclear but may be related to diabetic embryopathy [16] or perinatal complications. While our study detected risks for perinatal complications associated with different maternal glucose trajectories, we lacked power for formal mediation analyses.

### Methodology considerations for GBTM

GDM links to both short-term and long-term complications, making glucose trajectories vital as they offer insight into hyperglycemia’s timing, severity, and control. We used GBTM, a suitable tool that studies these patterns without trajectory distribution assumptions, given that such trajectories have never been studied before. Previous studies have reported that the selection of trajectory groups and population distribution across different classes were relatively robust across different software packages [55], though we only used a single software package. This model, frequently used in clinical research [43], recognizes different developmental courses of disease and does not assume a one-size-fits-all model. Such taxonomy primarily highlights differences in causes and outcomes of varying developmental trajectories, rather than implying entirely distinct population groups. However, GBTM has limitations [55, 56], such as assuming constant change rate and similar trajectory within a cluster. The Latent Growth Mixture Model (LGMM) offers another approach to identify trajectories, providing evaluation of individual-level variance within each trajectory, though it has more convergence issues and is more computationally demanding [47]. The choice between LGMM and GBTM depends on the scenario [57].

Vermunt et al. suggested a three-step approach to improve the prediction of class membership by incorporating misclassification errors in group membership [58]. To simplify, we used GEE models to investigate associations, adjusting for confounders and correcting for clustering of different children within the same mother instead of including outcomes and covariates in the GBTM and assessing risks of outcomes through post-estimation as would be done in a three-step approach. These models correct the standard error for clustering of different children within the same mother and are adjusted for potential confounders. However, our approach does not take misclassification errors into account during further analysis for different outcomes of interest, which is a limitation of this study and should be considered in future studies. A previous study demonstrated that the one-step approach is as efficient as the three-step method for model entropies of 0.6 or higher, with comparable results when entropy exceeds 0.80 [59]. We followed Nagin et al.’s criteria for model selection in GBTM [43], achieving a near-adequate fit with mismatch rates between 0.3 and 4.8%. The observed misclassification errors may drive associations towards null. However, the missing RCG values had minimal impact on model fitting.

### Strengths and limitations

This is the first study, to our knowledge, investigating glucose patterns throughout pregnancy and their related complications in mothers and children. We are unaware of any studies of risks of offspring NDCs in relation to maternal glucose measurements. Utilizing regional antenatal care records in our large register linkage, we obtained detailed information about maternal RCG levels and confounders collected prospectively relative to the outcomes, for a total population sample. While OGTT tests are the gold standard for insulin resistance, their longitudinal use at multiple antenatal visits is impractical. We used RCG instead, offering a continuous pregnancy-wide glucose variance rather than a single point measurement. While the quality and timing of the last food intake impact RCG levels, this was mitigated by standardizing RCG values. By excluding overweight or obese subjects in sensitivity analyses, we focused on hyperglycemia’s effects, disentangling the impact of overweight/obesity in associations.

Our findings should be interpreted in light of several limitations. Excluding women with RCG measurements missing in more than one time frame yielded a sample skewed towards Nordic-born mothers, prompting a need for replication in diverse populations to verify the generalizability of our findings. The study’s limited sample size prevented us from assessing confounding familial factors (e.g., genetics and environment) with sibling comparisons, as only 19% of the children had siblings born within the same range of years when data were available. Adjusting for maternal birth region [60], pregestational BMI [9, 12], and maternal psychiatric history as proxies for genetic liability has likely partially addressed these issues, though this remains a key issue to address in future studies.

In this study, we only included information on antidiabetic treatments to explain potential glycemic control in different trajectory groups but did not adjust for it because few women were treated with antidiabetic medications. Our inability to source information on nutritional or lifestyle interventions limited our interpretation of results, especially for groups who experienced elevated blood glucose levels followed by lower levels later in pregnancy. We suspect glucose drops are due to intentional control and interventions rather than physiological changes, given the increasing insulin resistance during pregnancy [61, 62].

While most obstetric/neonatal outcomes followed glucose variance identification, we could not determine the temporal relationship for some outcomes, like gestational hypertensive diseases. Even though hyperglycemia and hypertensive diseases might share common causes [63, 64], their causal relationship was not well-established. However, after excluding those with gestational hypertensive diseases, the associations between glucose trajectories and offspring NDCs were consistent with our primary findings.

While we set criteria for the minimum size of each trajectory group, linking the groups with proportionally fewer mothers to relatively rare NDC outcomes in children could introduce statistical power issues in assessing these associations. Some outcomes were broadly defined by combining sub-diagnoses, like obstructed labor due to various causes, as we lacked adequate power to investigate each sub-diagnosis. Despite children being of diagnosable age by the end of follow-up, the study’s follow-up time was less than ideal for register-based studies of NDCs, as past studies indicated lower NDC diagnoses in 7- to 9-year-olds compared to adolescents [6, 33]. Although the study detected an increased risk of perinatal complications and some NDC outcomes, it lacked the power for formal mediation analyses, suggesting the need for future studies with larger sample size.

## Conclusions

In summary, the associations between glucose trajectories indicating maternal hyperglycemia and the risks of short-term obstetric/neonatal complications and long-term NDCs in children depend on the severity, duration, timing of occurrence, and effective control of hyperglycemia during pregnancy. Our study highlights the importance of monitoring and controlling maternal glucose levels throughout pregnancy, as elevated levels at different stages may have different impacts on maternal and children’s outcomes. It also underscores the need for careful obstetric management in cases of moderately elevated glucose levels during pregnancy, which can complicate fetal growth and birth. Furthermore, the study highlights the importance of adopting individualized management approaches for women with different glucose trajectories during pregnancy, considering their specific risk profiles.

## Supplementary Information

**Additional file 1: Supplementary Methods & Table S1-S14 & Figure S1-S7.** Supplementary Methods-group-based trajectory modeling. The procedure involved in group-based trajectory modeling. **Table S1.** A description of random capillary glucose measurements. A description of random capillary glucose measurements during the entire pregnancy. **Table S2.** RCG values. Testing hour-standardized RCG z-scores and original RCG valuesmeasured at different times throughout the day. **Table S3.** Diagnostic codes. Diagnostic codes and register databases used to ascertain diagnoses within the Stockholm Youth Cohort. **Table S4.** Details of the selected group-based trajectory models. The details of the three best modelsfor each group-based trajectory model with different numbers of groups. **Table S5.** Model adequacy for selected models. The model adequacy of the 5-group and the 6-group model. **Table S6.** Characteristics of offspring born to mothers with or without missing RCG level data. Characteristics of offspring whose mothers had no missing values in random capillary glucose level, with 1missing value, or with more than 1 missing values in different time intervalsduring pregnancy. **Table S7.** Characteristics of offspring whose mothers have or do not have missing RCG level values within each time frame. Characteristics of offspring whose mothers had missing values in random capillary glucose levels during ≤20, >20-28, >28-34, and >34 wkGA throughout pregnancy. **Table S8.** Characteristics of the study sample. The Characteristics of the final study sample. **Table S9.** The parameter estimation. The parameter estimation of the final trajectory model. **Table S10.** The variance and covariance matrix. The variance and covariance matrix of the final trajectory model. **Table S11.** The misclassification error matrix. The misclassification error matrix of the final trajectory model. **Table S12.** Statistics for model adequacy. Statistics for the adequacy of the final trajectory model, considering scenarios where missing values were replaced with either the smallest or largest RCG levels within each time frame, or where instances with missing values were excluded altogether. **Table S13.** Sensitivity analysis by excluding women with overweight/obesity. The sensitivity analysis of the association between trajectory groups and obstetric/neonatal outcomes and offspring NDCs outcomes by excluding women with overweight/obesity. **Table S14.** Sensitivity analysis by excluding gestational hypertensive diseases. Sensitivity analysis for the association between glucose trajectories and offspring NDCs by excluding gestational hypertensive diseases. **Fig S1. **Sample derivation. A description of sample derivation. **Fig S2.** A depiction of RCG levels measured at different times over a day. Median, 25^th^ and 75^th^ percentile of random capillary glucose levelsmeasured at different times from 07:00 to <19:00 throughout pregnancy. **Fig S3.** The distribution of average RCG z-scores throughout pregnancy. Histograms illustrating the distribution of RCG z-scores in general, as well as during each time frame throughout pregnancy. **Fig S4.** Overlapping and mutually exclusive NDC outcomes. A description of NDC outcomes, accounting for both instances of overlap and non-overlap in the final study sample. **Fig S5.** The BIC values for each model. A depiction of BIC values for each trajectory model consisting of 1-6 groups and featuring polynomials ranging from 1 to 3. **Fig S6.** The best-fitting 5-group and the best-fitting 6-group models. A depiction of the best-fitting 5-groupand the best-fitting 6-group models. **Fig S7.** RCG z-score trajectory groups in the sensitivity analyses. RCG z-score trajectory groups after excluding those with RCG missing in any time intervals, replacing missing values with the smallest, and largest values during each time interval in pregnancy.

**Additional file 2.** GRoLTS checklist. Guidelines for Reporting on Latent Trajectory Studieswere adhered to bolster our reporting on the Group-Based Trajectory Modeling.

**Additional file 3.** STROBE checklist. The STROBE checklist showing our study was reported according to the STROBE checklist for cohort studies.

## Data Availability

Data used for the current study were anonymized and obtained from Statistics Sweden and the National Board of Health and Welfare after ethical and legal assessment. Researchers interested in obtaining the data and replicating our results can make inquiry through these data holders. For further information, see https://www.scb.se/en/services/guidance-for-researchers-and-universities/.

## Abbreviations

ADHD: Attention-deficit/hyperactivity disorders
AGA: Appropriate for gestational age
BMI: Body mass index
CI: Confidence interval
GBTM: Group-based trajectory modeling
GDM: Gestational diabetes mellitus
GWG: Gestational weight gain
ICD: International Classification of Diseases
ID: Intellectual disability
IQR: Interquartile rang
LGA: Large for gestational age
NDC: Neurodevelopmental conditions
OGTT: Oral glucose tolerance test
OR: Odds ratio
RGWG-T2: Rate of gestational weight gain in the second trimester
RGWG-T3: Rate of gestational weight gain in the third trimester
RPG: Random capillary glucose
SD: Standard deviation
SGA: Small for gestational age
wkGA: Weeks of gestational age

## References

[1] Ye W, Luo C, Huang J, Li C, Liu Z, Liu F (2022). Gestational diabetes mellitus and adverse pregnancy outcomes: systematic review and meta-analysis. BMJ.

[2] Santos S, Voerman E, Amiano P, Barros H, Beilin LJ, Bergström A (2019). Impact of maternal body mass index and gestational weight gain on pregnancy complications: an individual participant data meta-analysis of European North American and Australian cohorts. BJOG.

[3] Billionnet C, Mitanchez D, Weill A, Nizard J, Alla F, Hartemann A (2017). Gestational diabetes and adverse perinatal outcomes from 716,152 births in France in 2012. Diabetologia.

[4] Riskin A, Itzchaki O, Bader D, Iofe A, Toropine A, Riskin-Mashiah S (2020). Perinatal outcomes in infants of mothers with diabetes in pregnancy. Isr Med Assoc J.

[5] Metzger BE, Lowe LP, Dyer AR, Trimble ER, Chaovarindr U, HAPO Study Cooperative Research Group (2008). Hyperglycemia and adverse pregnancy outcomes. N Engl J Med.

[6] Chen S, Zhao S, Dalman C, Karlsson H, Gardner R (2021). Association of maternal diabetes with neurodevelopmental disorders: autism spectrum disorders, attention-deficit/hyperactivity disorder and intellectual disability. Int J Epidemiol.

[7] Xiang AH, Wang X, Martinez MP, Walthall JC, Curry ES, Page K (2015). Association of maternal diabetes with autism in offspring. JAMA.

[8] Gardener H, Spiegelman D, Buka SL (2009). Prenatal risk factors for autism: a comprehensive meta-analysis. Br J Psychiatry.

[9] Li M, Fallin MD, Riley A, Landa R, Walker SO, Silverstein M (2016). The association of maternal obesity and diabetes with autism and other developmental disabilities. Pediatrics.

[10] Wan H, Zhang C, Li H, Luan S, Liu C (2018). Association of maternal diabetes with autism spectrum disorders in offspring: a systemic review and meta-analysis. Medicine (Baltimore).

[11] Xu G, Jing J, Bowers K, Liu B, Bao W (2014). Maternal diabetes and the risk of autism spectrum disorders in the offspring: a systematic review and meta-analysis. J Autism Dev Disord.

[12] Girchenko P, Tuovinen S, Lahti-Pulkkinen M, Lahti J, Savolainen K, Heinonen K (2018). Maternal early pregnancy obesity and related pregnancy and pre-pregnancy disorders: associations with child developmental milestones in the prospective PREDO Study. Int J Obes (Lond).

[13] Xiang AH, Wang X, Martinez MP, Getahun D, Page KA, Buchanan TA (2018). Maternal gestational diabetes mellitus, type 1 diabetes, and type 2 diabetes during pregnancy and risk of ADHD in offspring. Diabetes Care.

[14] Zhao L, Li X, Liu G, Han B, Wang J, Jiang X (2019). The association of maternal diabetes with attention deficit and hyperactivity disorder in offspring: a meta-analysis. Neuropsychiatr Dis Treat.

[15] Rowland J, Wilson CA (2021). The association between gestational diabetes and ASD and ADHD: a systematic review and meta-analysis. Sci Rep.

[16] Márquez-Valadez B, Valle-Bautista R, García-López G, Díaz NF, Molina-Hernández A (2018). Maternal diabetes and fetal programming toward neurological diseases: beyond neural tube defects. Front Endocrinol.

[17] Wickström R, Skiöld B, Petersson G, Stephansson O, Altman M (2018). Moderate neonatal hypoglycemia and adverse neurological development at 2–6 years of age. Eur J Epidemiol.

[18] Lord C, Elsabbagh M, Baird G, Veenstra-Vanderweele J (2018). Autism spectrum disorder. Lancet.

[19] Faraone SV, Asherson P, Banaschewski T, Biederman J, Buitelaar JK, Ramos-Quiroga JA (2015). Attention-deficit/hyperactivity disorder. Nat Rev Dis Primers.

[20] Huang J, Zhu T, Qu Y, Mu D (2016). Prenatal, perinatal and neonatal risk factors for intellectual disability: a systemic review and meta-analysis. PLoS ONE.

[21] Bilder DA, Pinborough-Zimmerman J, Bakian AV, Miller JS, Dorius JT, Nangle B (2013). Prenatal and perinatal factors associated with intellectual disability. Am J Intellect Dev Disabil.

[22] Farrar D, Duley L, Dowswell T, Lawlor DA (2017). Different strategies for diagnosing gestational diabetes to improve maternal and infant health. Cochrane Database Syst Rev.

[23] International Association of Diabetes and Pregnancy Study Groups Consensus Panel, Metzger BE, Gabbe SG, Persson B, Buchanan TA, Catalano PA, Damm P, Dyer AR, Leiva Ad, Hod M, Kitzmiler JL, Lowe LP, McIntyre HD, Oats JJ, Omori Y, Schmidt MI. International association of diabetes and pregnancy study groups recommendations on the diagnosis and classification of hyperglycemia in pregnancy. Diabetes Care. 2010;33(3):676–82. 10.2337/dc09-1848 PMC2827530 20190296

[24] Cosson E, Benbara A, Pharisien I, Nguyen MT, Revaux A, Lormeau B (2013). Diagnostic and prognostic performances over 9 years of a selective screening strategy for gestational diabetes mellitus in a cohort of 18,775 subjects. Diabetes Care.

[25] Nwali SA, Onoh RC, Dimejesi IB, Obi VO, Jombo SE, Edenya OO (2021). Universal versus selective screening for gestational diabetes mellitus among antenatal clinic attendees in Abakaliki: using the one-step 75 gram oral glucose tolerance test. BMC Pregn Childb.

[26] Danilenko-Dixon DR, Van Winter JT, Nelson RL, Ogburn PL (1999). Universal versus selective gestational diabetes screening: application of 1997 American diabetes association recommendations. Am J Obstet Gynecol.

[27] Recommendations | Diabetes in pregnancy: management from preconception to the postnatal period | Guidance | NICE. https://www.nice.org.uk/guidance/ng3/chapter/Recommendations#gestational-diabetes. Accessed 12 Apr 2022.

[28] Feig DS, Berger H, Donovan L, Godbout A, Kader T, Diabetes Canada Clinical Practice Guidelines Expert Committee (2018). Diabetes and pregnancy. Can J Diabetes.

[29] González-Quintero VH, Istwan NB, Rhea DJ, Rodriguez LI, Cotter A, Carter J (2007). The impact of glycemic control on neonatal outcome in singleton pregnancies complicated by gestational diabetes. Diabetes Care.

[30] Yogev Y, Xenakis EMJ, Langer O (2004). The association between preeclampsia and the severity of gestational diabetes: the impact of glycemic control. Am J Obstet Gynecol.

[31] Lindqvist M, Persson M, Lindkvist M, Mogren I (2014). No consensus on gestational diabetes mellitus screening regimes in Sweden: pregnancy outcomes in relation to different screening regimes 2011 to 2012, a cross-sectional study. BMC Pregnancy Childbirth.

[32] Johansson K, Hutcheon JA, Stephansson O, Cnattingius S (2016). Pregnancy weight gain by gestational age and BMI in Sweden: a population-based cohort study. Am J Clin Nutr.

[33] Idring S, Lundberg M, Sturm H, Dalman C, Gumpert C, Rai D (2015). Changes in prevalence of autism spectrum disorders in 2001–2011: findings from the Stockholm youth cohort. J Autism Dev Disord.

[34] Association AD (2004). Hospital admission guidelines for diabetes*. Diabetes Care.

[35] Diabetes i samband med graviditet. https://kunskapsstodforvardgivare.se/omraden/kvinnosjukdomar-och-forlossning/riktlinjer-for-bmm/barnmorskemottagning/graviditet/riktlinjer/diabetes-i-samband-med-graviditet. Accessed 12 Jun 2022.

[36] ACOG Practice Bulletin No (2018). 190: Gestational diabetes mellitus. Obstet Gynecol.

[37] Idring S, Rai D, Dal H, Dalman C, Sturm H, Zander E (2012). Autism spectrum disorders in the Stockholm youth cohort: design, prevalence and validity. PLoS ONE.

[38] Kosidou K, Dalman C, Widman L, Arver S, Lee BK, Magnusson C (2017). Maternal polycystic ovary syndrome and risk for attention-deficit/hyperactivity disorder in the offspring. Biol Psychiatry.

[39] Hu J, Ge Z, Xu Q, Shen S, Wang Y, Zhu D (2020). Influence of fetal sex on perinatal outcomes in women with gestational diabetes mellitus. Diabetes Metab Res Rev.

[40] Gudmundsson S, Björgvinsdóttir L, Molin J, Gunnarsson G, Marsal K (1997). Socioeconomic status and perinatal outcome according to residence area in the city of Malmö. Acta Obstet Gynecol Scand.

[41] Yuen L, Wong VW (2015). Gestational diabetes mellitus: challenges for different ethnic groups. World J Diabetes.

[42] Nguena Nguefack HL, Pagé MG, Katz J, Choinière M, Vanasse A, Dorais M (2020). Trajectory modelling techniques useful to epidemiological research: a comparative narrative review of approaches. Clin Epidemiol.

[43] Nagin DS, Odgers CL (2010). Group-based trajectory modeling in clinical research. Annu Rev Clin Psychol.

[44] Basprogram för vård under graviditet. Stockholms läns landsting Maj PDF Gratis nedladdning. https://docplayer.se/6915353-Basprogram-for-vard-under-graviditet-stockholms-lans-landsting-maj-2011.html. Accessed 10 Mar 2023.

[45] Rani PR, Begum J (2016). Screening and diagnosis of gestational diabetes mellitus, where do we stand. J Clin Diagn Res.

[46] Nagin DS (2005). Group-based modeling of development.

[47] van de Schoot R, Sijbrandij M, Winter SD, Depaoli S, Vermunt JK (2017). The GRoLTS-checklist: guidelines for reporting on latent trajectory studies. Struct Equ Modeling.

[48] Benjamini Y, Hochberg Y (1995). Controlling the false discovery rate: a practical and powerful approach to multiple testing. J Roy Stat Soc: Ser B (Methodol).

[49] Sermer M, Naylor CD, Gare DJ, Kenshole AB, Ritchie JWK, Farine D (1995). Impact of increasing carbohydrate intolerance on maternal-fetal outcomes in 3637 women without gestational diabetes: The Toronto tri-hospital gestational diabetes project. Am J Obstet Gynecol.

[50] Priest JR, Yang W, Reaven G, Knowles JW, Shaw GM (2015). Maternal midpregnancy glucose levels and risk of congenital heart disease in offspring. JAMA Pediatr.

[51] Zou J, Wei Q, Shi Y, Wang K, Zhang Y, Shi H (2022). Longitudinal associations between maternal glucose levels and ultrasonographic fetal biometrics in a Shanghai cohort. JAMA Netw Open.

[52] Han S, Crowther CA, Middleton P (2012). Interventions for pregnant women with hyperglycaemia not meeting gestational diabetes and type 2 diabetes diagnostic criteria. Cochrane Database Syst Rev.

[53] Metzger BE, Gabbe SG, Persson B, Buchanan TA, Catalano PA, International Association of Diabetes and Pregnancy Study Groups Consensus Panel (2010). International association of diabetes and pregnancy study groups recommendations on the diagnosis and classification of hyperglycemia in pregnancy. Diabetes Care.

[54] Desoye G, Nolan CJ (2016). The fetal glucose steal: an underappreciated phenomenon in diabetic pregnancy. Diabetologia.

[55] Serra L, Farrants K, Alexanderson K, Ubalde M, Lallukka T (2022). Trajectory analyses in insurance medicine studies: examples and key methodological aspects and pitfalls. PLoS ONE.

[56] Herle M, Micali N, Abdulkadir M, Loos R, Bryant-Waugh R, Hübel C (2020). Identifying typical trajectories in longitudinal data: modelling strategies and interpretations. Eur J Epidemiol.

[57] Mésidor M, Rousseau M-C, O’Loughlin J, Sylvestre M-P (2022). Does group-based trajectory modeling estimate spurious trajectories?. BMC Med Res Methodol.

[58] Vermunt JK (2017). Latent class modeling with covariates: two improved three-step approaches. Polit Anal.

[59] Asparouhov T, Muthén B (2014). Auxiliary variables in mixture modeling: three-step approaches using Mplus. Struct Equ Modeling.

[60] Hedderson MM, Darbinian JA, Ferrara A (2010). Disparities in the risk of gestational diabetes by race-ethnicity and country of birth. Paediatr Perinat Epidemiol.

[61] Kirwan JP, Hauguel-De Mouzon S, Lepercq J, Challier J-C, Huston-Presley L, Friedman JE (2002). TNF-α is a predictor of insulin resistance in human pregnancy. Diabetes.

[62] Ryan EA (2003). Hormones and insulin resistance during pregnancy. The Lancet.

[63] Hedderson MM, Ferrara A (2008). High blood pressure before and during early pregnancy is associated with an increased risk of gestational diabetes mellitus. Diabetes Care.

[64] Lao TT, Ho L-F (2003). First-trimester blood pressure and gestational diabetes in high-risk Chinese women. J Soc Gynecol Investig.

