# Supplementary material for: Random capillary glucose levels throughout pregnancy, obstetric and neonatal outcomes, and long-term neurodevelopmental conditions in children: a group-based trajectory analysis
Source: BMC Med. 2023 Jul 19;21:260. doi: 10.1186/s12916-023-02926-3 (PMC10354916; doi:10.1186/s12916-023-02926-3)
Supplement: Supplementary file 1 — Additional file 1: Supplementary Methods & Table S1-S14 & Figure S1-S7. Supplementary Methods-group-based trajectory modeling. The procedure involved in group-based trajectory modeling. Table S1. A description of random capillary glucose measurements. A description of random capillary glucose measurements during the entire pregnancy. Table S2. RCG values. Testing hour-standardized RCG z-scores and original RCG valuesmeasured at different times throughout the day. Table S3. Diagnostic codes. Diagnostic codes and register databases used to ascertain diagnoses within the Stockholm Youth Cohort. Table S4. Details of the selected group-based trajectory models. The details of the three best modelsfor each group-based trajectory model with different numbers of groups. Table S5. Model adequacy for selected models. The model adequacy of the 5-group and the 6-group model. Table S6. Characteristics of offspring born to mothers with or without missing RCG level data. Characteristics of offspring whose mothers had no missing values in random capillary glucose level, with 1missing value, or with more than 1 missing values in different time intervalsduring pregnancy. Table S7. Characteristics of offspring whose mothers have or do not have missing RCG level values within each time frame. Characteristics of offspring whose mothers had missing values in random capillary glucose levels during ≤20, >20-28, >28-34, and >34 wkGA throughout pregnancy. Table S8. Characteristics of the study sample. The Characteristics of the final study sample. Table S9. The parameter estimation. The parameter estimation of the final trajectory model. Table S10. The variance and covariance matrix. The variance and covariance matrix of the final trajectory model. Table S11. The misclassification error matrix. The misclassification error matrix of the final trajectory model. Table S12. Statistics for model adequacy. Statistics for the adequacy of the final trajectory model, considering scenarios where missin [file 12916_2023_2926_MOESM1_ESM.docx]

**Additional file 1**

**Supplementary Methods**

**Step 1: Data preparation for GBTM (including determining the metric of time)**

According to the guidelines for antenatal care in the Stockholm region's “BASPROGRAM FÖR VÅRD UNDER GRAVIDITET (2011)”, random capillary glucose levels ("p-glukos") should be measured five times during pregnancy: during the first antenatal care visit (around 10-12 weeks of gestation [wkGA]) and at 25, 29, 33, and 37 wkGA (Ref 44 in the main text).

According to **Additional file 1 (Table S1A)**, in our study sample, the median p-glukos measurement time is 5.0 (IQR 4.0-6.0), and the median gestational week of the first "p-glukos" measurement is week 10 (IQR 9.0-11.0). These values are in line with the guideline. Clinically speaking, glucose measurements may not align exactly with the recommended week according to guidelines. Instead, it is reasonable to measure around the suggested gestational weeks. Additionally, clinicians/midwives may require multiple measurements based on their empirical experiences with a particular pregnant woman.

The numbers highlighted in yellow in the second table below can explain why we used four time intervals instead of five. During the >28-34 wkGA period, 46.43% of the population had only one measurement, while 48.32% had at least two measurements. Separating this period into two intervals would generate a large number of missing values for each of the newly generated intervals.

Our data are unstructured in terms of time, as p-glukos levels were measured at different gestational weeks for different women. As shown in **Additional file 1, Table S1B**, more than half of the women measured p-glukos only once during ≤20, >20-28, and >34 wkGA. During >28-34 wkGA, about half of them measured p-glukos once and the other half measured it at least twice. To simplify the modeling, we calculated the average RCG z-scores for each time interval during pregnancy.

We also checked the median gestational week differences between the first and last measurement of RCG if there were more than one measurement during time intervals of ≤20, 20-28, >28-34, and >34 wkGA (i.e., 9 [IQR 7-11]; 4 [IQR 3-4]; 3 [IQR 3-4]; 3 [IQR 2-4] for each interval). Although we found large gestational week differences between the first and last RCG measurement among those who measured RCG at least twice before 20 wkGA, it accounted for only about 15% of the population (**Additional file 1, Table S1B**). In addition, glucose levels in early pregnancy (first trimester and early second trimester) are less likely to be influenced by pregnancy (fasting and postprandial glucose concentrations are normally lower than in normal non-pregnant women); instead, they mainly reflect a pre-gestational state (Ref 45 in the main text).

To simplify modeling, we calculated the average RCG z-scores for each time interval during pregnancy. In other words, we reorganized the dataset into a structured metric of time and used average RCG z-scores during each time interval (if multiple measurements were conducted) as the corresponding values.

**Step 2. Model selection**

Our preliminary model selection was based on Bayesian information criteria (BIC) values. According to Nagin et al., Bayesian Information Criterion (BIC) is calculated by log(*L*)-0.5*k* log(*N*). In Stata (traj plugin), models with larger BIC scores (less negative) are considered better using Stata “traj” plugin (Ref 46 in the main text). We first used loop programming to assess the models using BIC after fitting different permutations of polynomials (from linear, quadratic to cubic and from one to six groups), generating a total of 1,092 models. The BIC values for each model are depicted in **Additional file 1, Fig S5.** As shown in the figure, BIC values sharply increased when the number of groups increased from 1 to 5, and then increased mildly and steadily when the group numbers increased from 5 to 6.

We present the details of the three best models (with the highest BIC values) for each group-based trajectory model with different numbers of groups (all the following models have converged) (**Additional file 1, Table S4**). Based on the BIC and entropy values, the 6-group trajectory model is a superior model. However, we expected each trajectory to contain at least 1000 individuals to facilitate further analysis. In the 6-group GBTMs, there is always one group that contains too few observations (less than 1000).

We presented the plotting figures of the best-fitting 5-group (polynomials 3 3 3 3 3) and the best-fitting 6-group models (polynomials 3 3 3 3 3 3) in **Additional file 1, Fig S6**.

According to **Additional file 1, Fig S6**, the main difference between the 5-group model (5 cubic trajectories) and the 6-group model (6 cubic trajectories) was that the 6-group model separated the 5th group in the 5-group model into two different trajectories. One trajectory had a peak during >28-34 wkGA, while the other had persistently high glucose levels with a peak during >34 wkGA. However, the 5th and 6th groups in the 6-group model mainly reflected a peak of glucose levels in late pregnancy, which could also be observed in the 5th group of the 5-group model (i.e., persistently high glucose levels throughout pregnancy with a peak in early third trimester).

We also compared the model adequacy of the 5-group and the 6-group model (**Additional file 1, Table S5**). The 6-group cubic model did not address the issue of low odds of correct classification (below 5) in Group 1 that was present in the 5-group cubic model. Instead, it introduced another issue: the average posterior probability in Group 2 was lower than 0.70.

Considering multiple perspectives, we have decided to proceed with further analysis using the 5-group cubic model.

**Step 3: To achieve the global maxima**

We chose the 5-group models (polynomials 3 3 3 3 3) for further analysis. To achieve the global maxima, we rerun the model with a matrix of random start values (variability of 0.002) and 10000 iterations.

**Step 4. Check the parameter estimations and the variance-covariance matrix**

Parameter estimations were presented in **Additional file 1, Table S9** and **Additional file 1, Table S10**.

**Step 5: Check the misclassification errors**

The misclassification error matrix for the final trajectory model (with 5 cubic trajectories) was presented in **Additional file 1, Table S11**.

**Step 6: Check the model adequacy**

The table for the model adequacy was presented in **Additional file 1, Table S12**.

**Step 7: Check the influence of missing values**

In the sensitivity analyses, we replicated the GBTM by excluding those with missing RCG values or replacing the missing values with the smallest or the largest RCG values in each time interval. We plotted the trajectory groups in **Additional file 1, Fig S7,** and presented the model adequacy in **Additional file 1, Table S12**.

**Fig S1. Study sample derivation.** We included children born in Sweden between 2007-2010 with information from both OBSTETRIX and the Medical Birth Register (MBR). The study cohort was nested in the Stockholm Youth Cohort (SYC). ^a^ Children born before 2007 were excluded from the study population since OBSTETRIX data collection began in 2007. ^b^Children born in 2011 were excluded from the study population since the Medical Birth Register (MBR) data from 2011 was not yet available at the time of register linkage. **Abbreviations:** RCG- Random capillary glucose


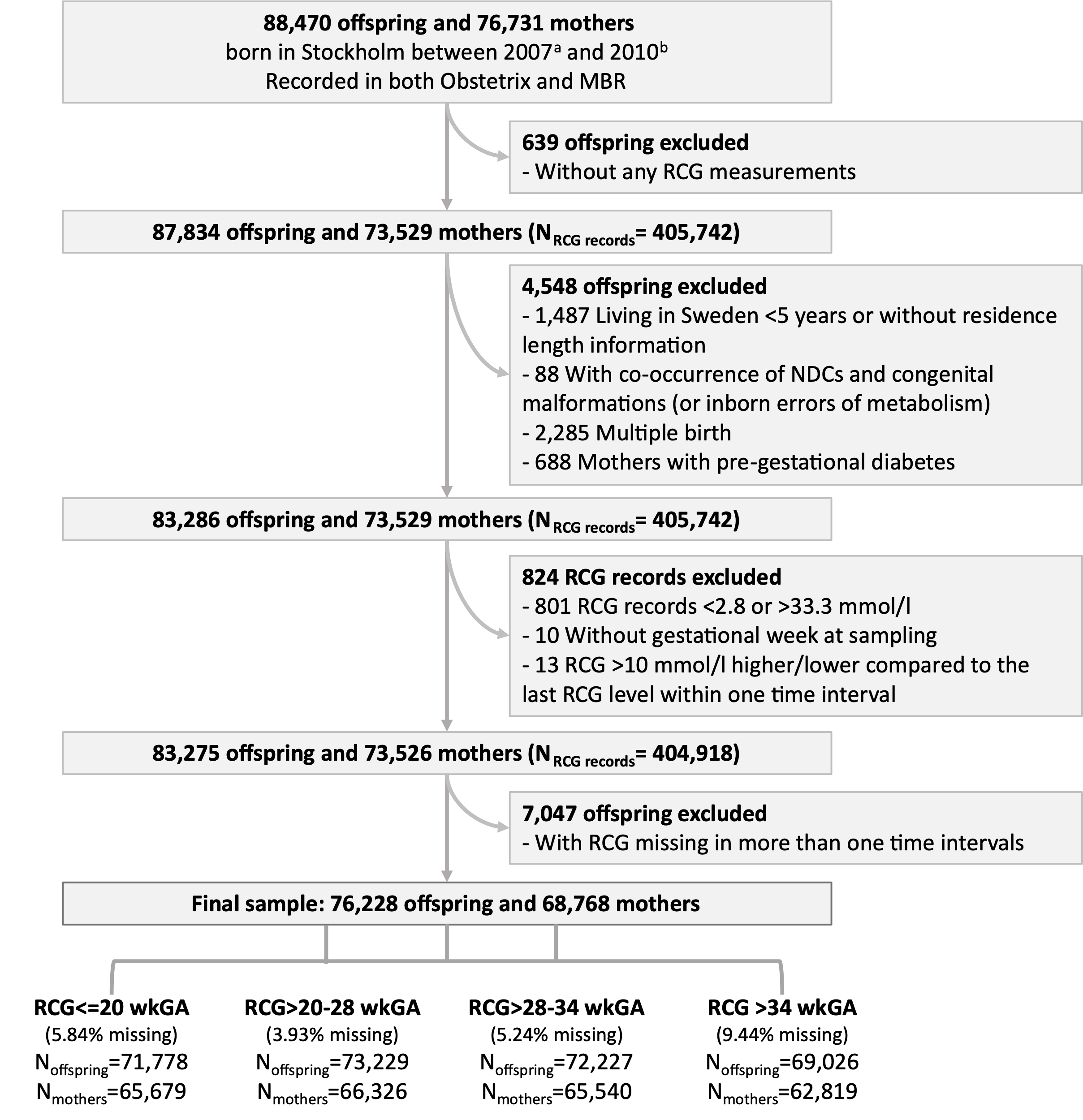


**Fig S2.** Median, 25^th^ and 75^th^ percentile of random capillary glucose levels (mmol/l) measured at different times from 07:00 to <19:00 throughout pregnancy.


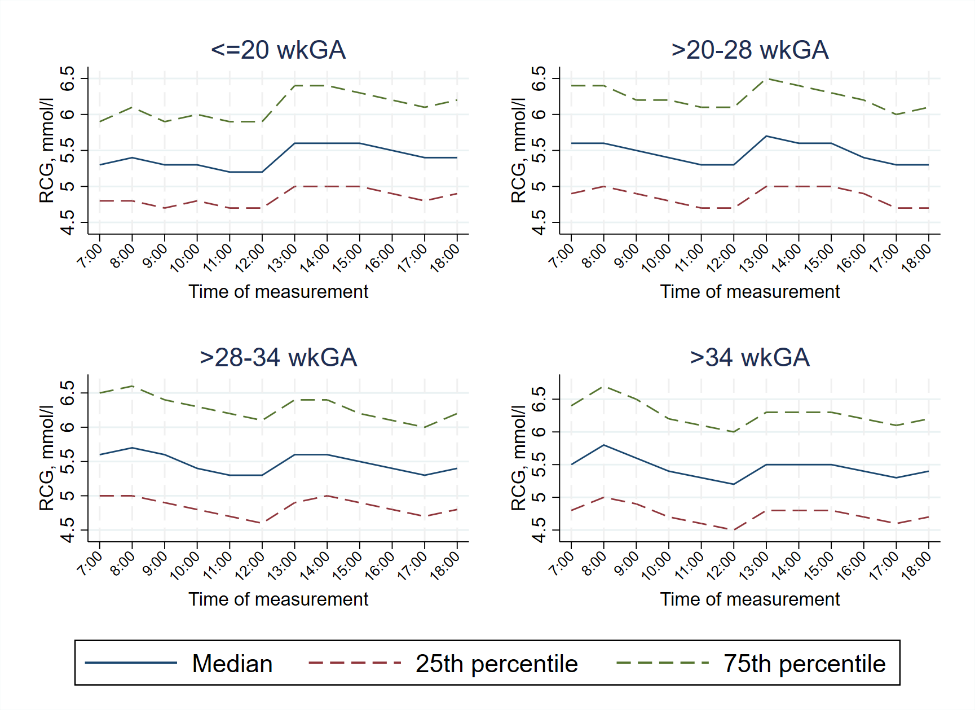


**Fig S3.** The distribution of average RCG z-scores throughout pregnancy. (Panel A) We transformed RCG levels (mmol/l) into z-scores according to the time of measurement over a day (07:00-18:00) by using formula: (RCG-mean (RCG))/sd (RCG) for each measurement hour. (Panel B) An average RCG z-score was calculated for each interval during pregnancy (i.e., ≤20, >20-28, >28-34, >34 wkGA).


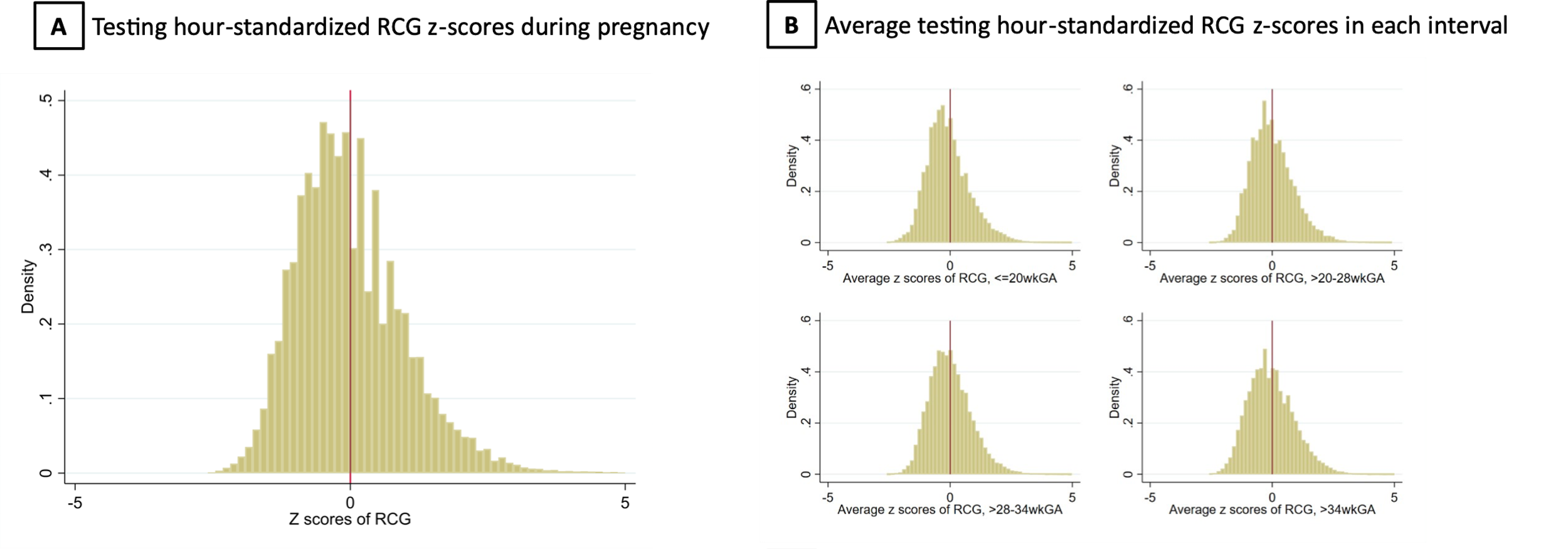


**Fig S4.** Overlapping and mutually exclusive NDC outcomes.

**
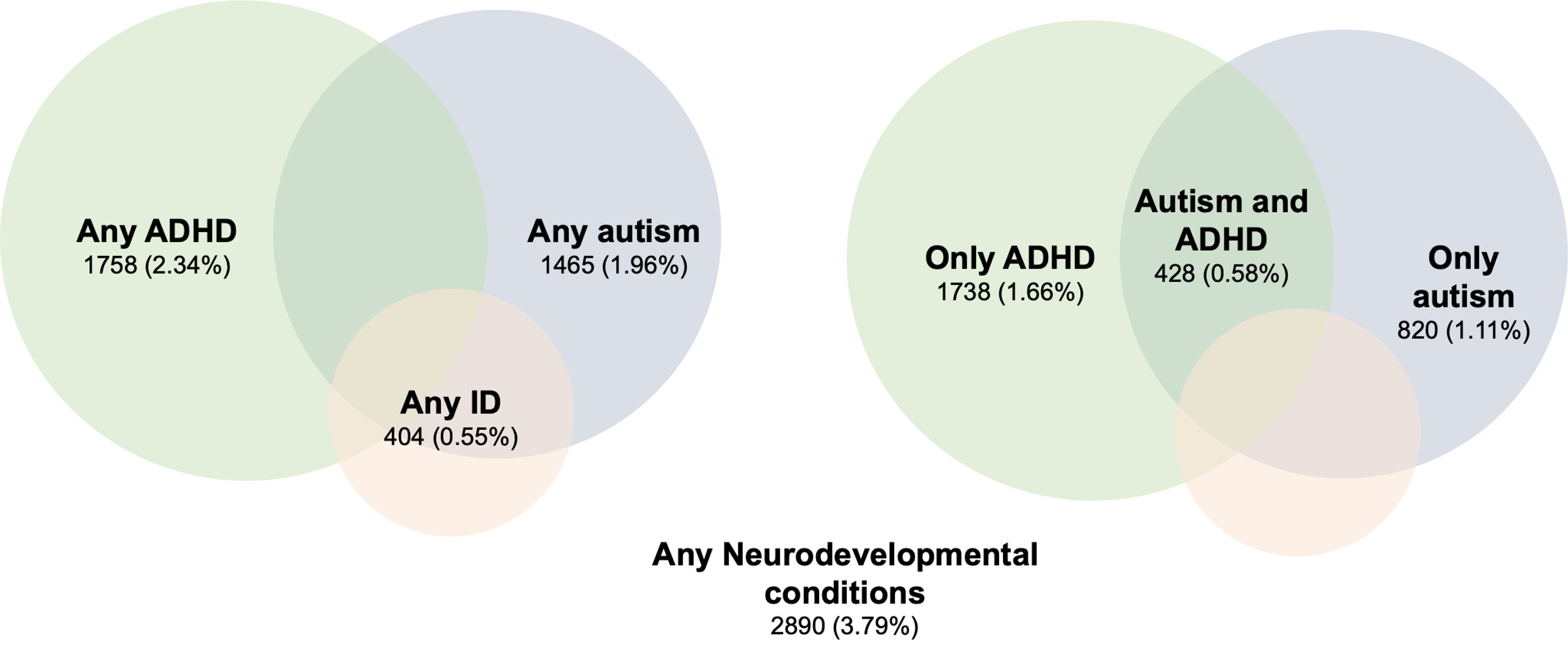
**

**Fig S5.** The BIC values for each model

**Fig S6.** The best-fitting 5-group (polynomials 3 3 3 3 3) and the best-fitting 6-group models (polynomials 3 3 3 3 3 3).

1. 5-group cubic GBTM (polynomials 3 3 3 3 3)


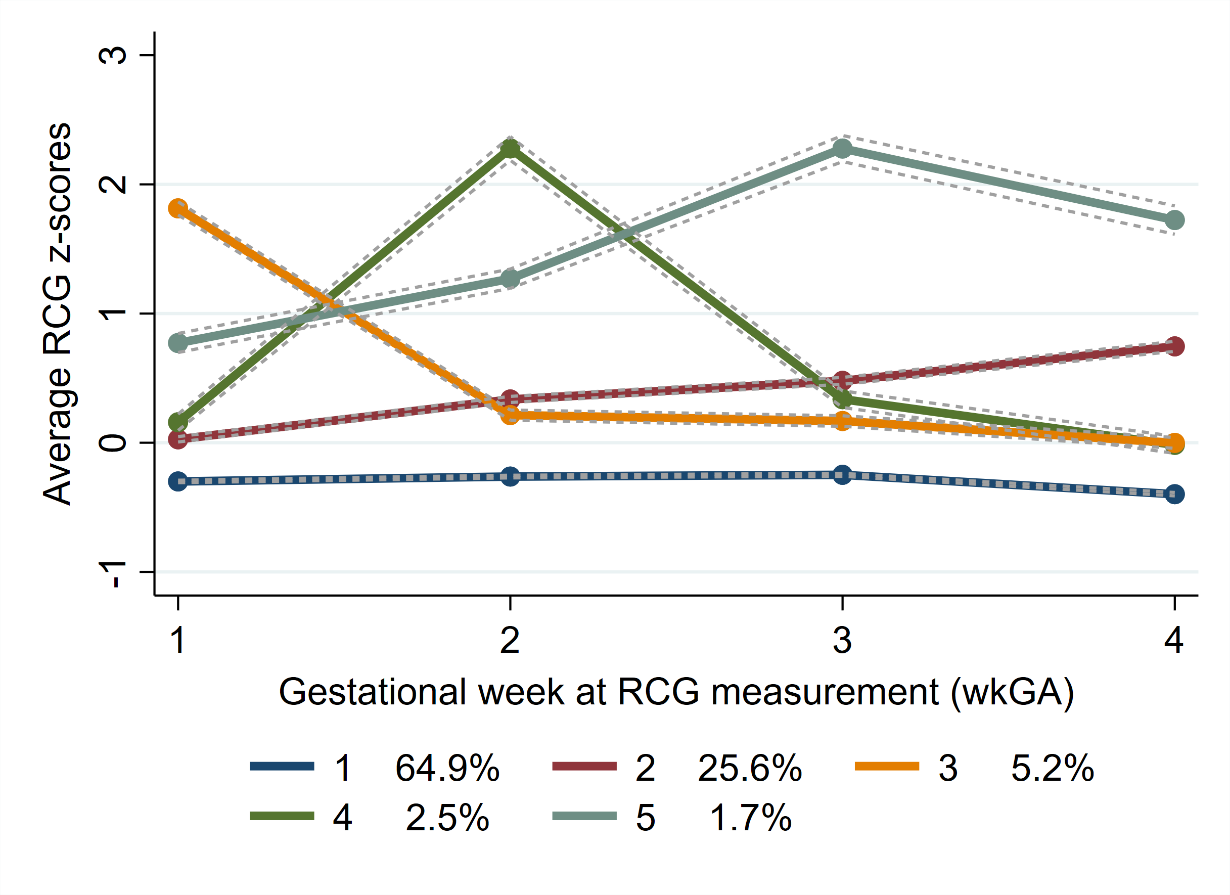


1. 6-group cubic GBTM (polynomials 3 3 3 3 3 3)


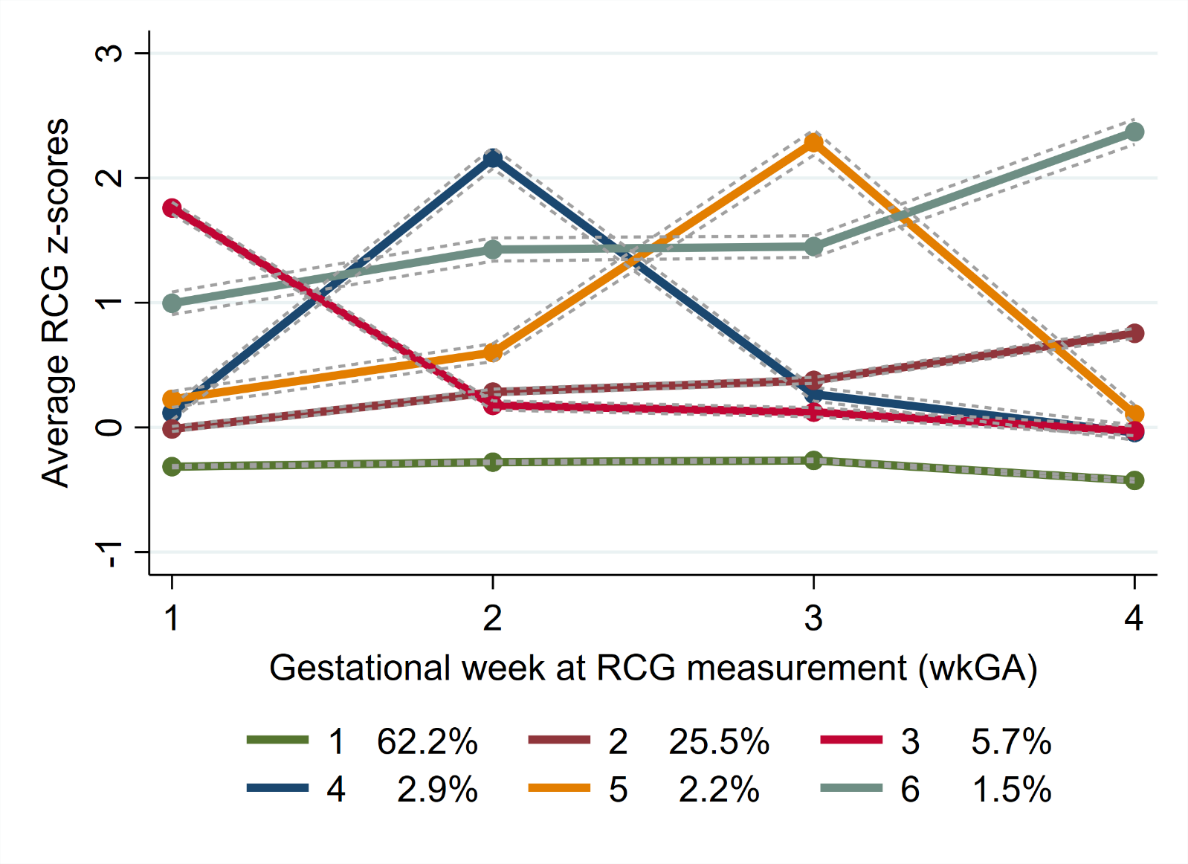


**Fig S7.** RCG z-score trajectory groups after excluding those with RCG missing in any time intervals (N_excluded_=18,652), replacing missing values with the smallest, and largest values during each time interval in pregnancy

1. GBTM in the full cohort (N=76,228)


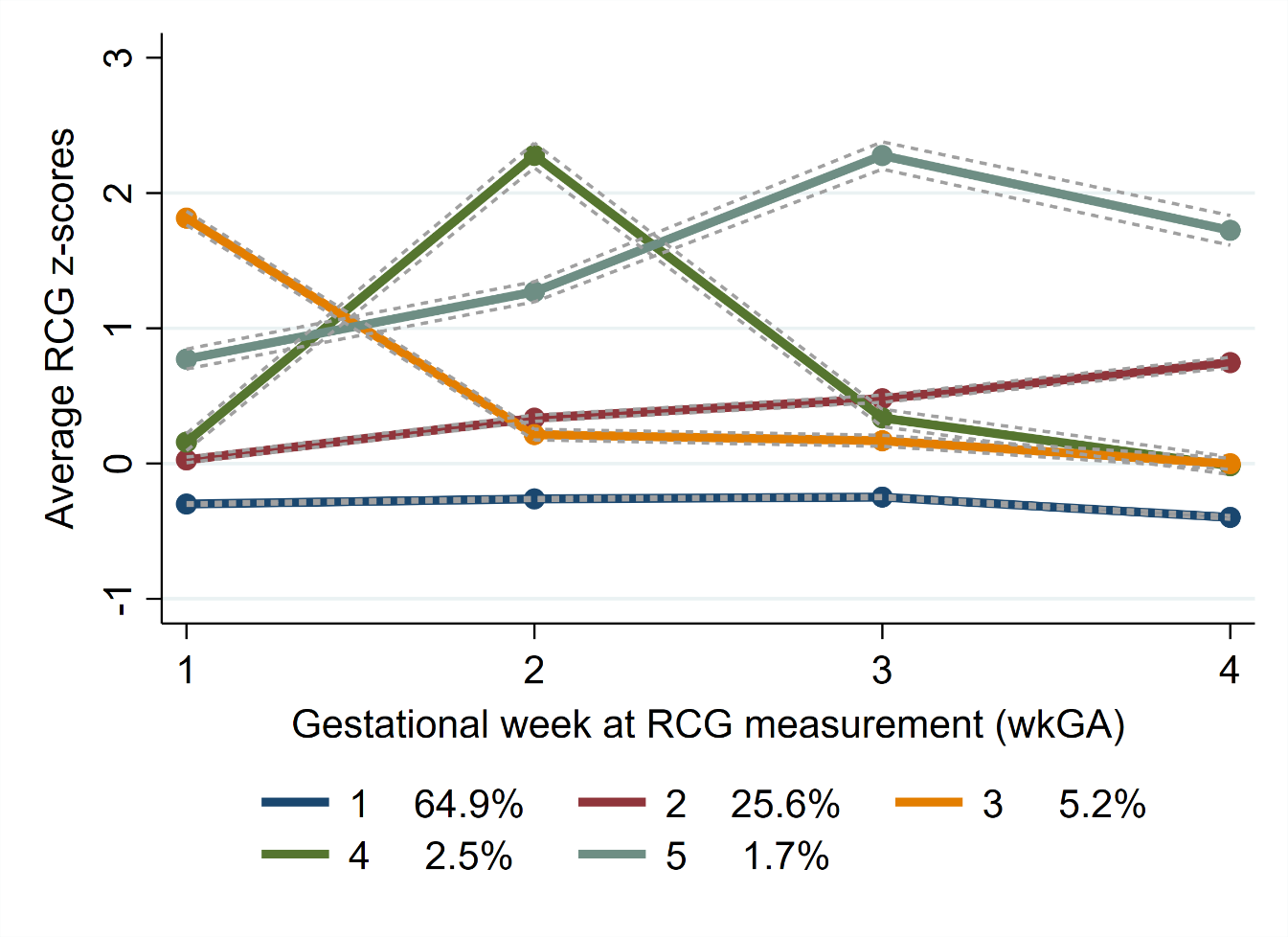


1. GBTM after excluding missing values in any time intervals (N_excluded_=18,652)


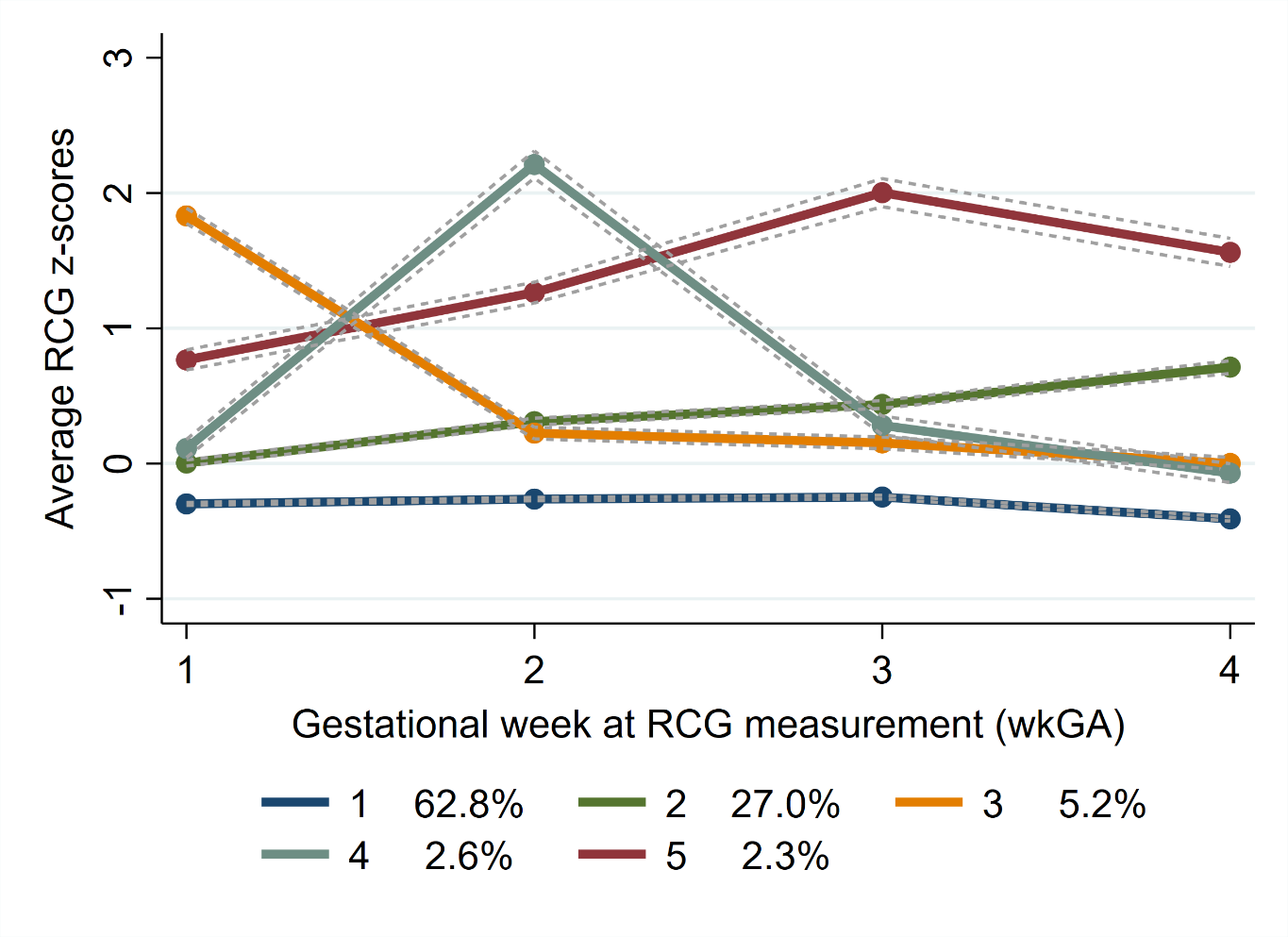


1. GBTM after replacing missing values with the smallest value in each time interval (N=76,228)


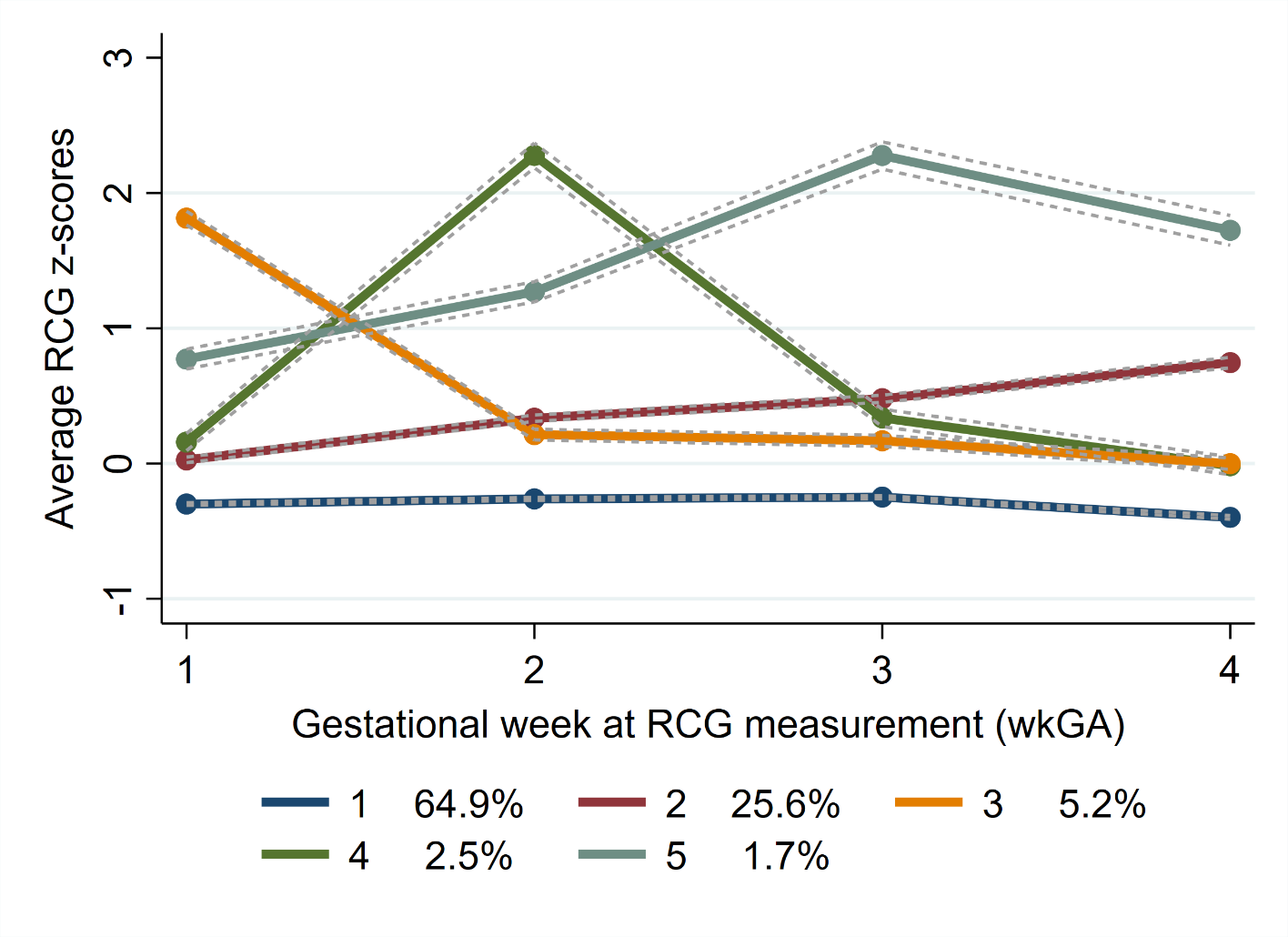


1. GBTM after replacing missing values with the largest value in each time interval (N=76,228)


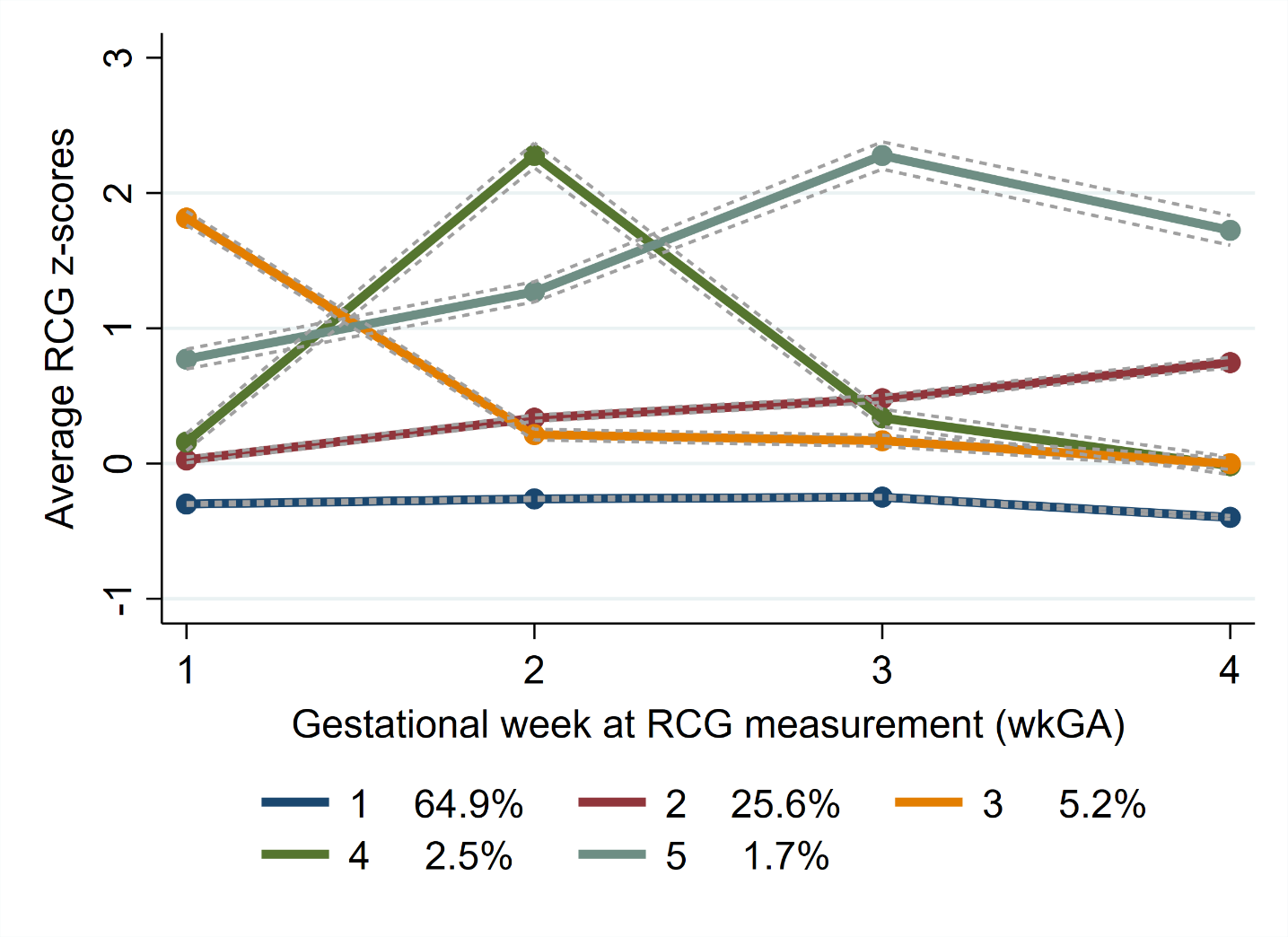


Table S1. A description of random capillary glucose measurements during the entire pregnancy

1. General description

| **Characteristics, median (IQR)** |  |
| --- | --- |
| **Total** | 76228 |
| **RCG measurements during the entire pregnancy (times)** | 5.0 (4.0, 6.0) |
| **RCG measurements for each time interval (times)** |  |
| ≤20 wkGA | 1.0 (1.0, 1.0) |
| >20-28 wkGA | 1.0 (1.0, 2.0) |
| >28-34 wkGA | 2.0 (1.0, 2.0) |
| >34 wkGA | 1.0 (1.0, 2.0) |
| **The first RCG measurement timing** **for each time interval (wkGA)** |  |
| ≤20 wkGA | 10.0 (9.0, 11.0) |
| >20-28 wkGA | 25.0 (24.0, 25.0) |
| >28-34 wkGA | 30.0 (29.0, 32.0) |
| >34 wkGA | 37.0 (36.0, 37.0) |
| **The last RCG measurement timing** **for each time interval (wkGA)** |  |
| ≤20 wkGA | 11.0 (9.0, 13.0) |
| >20-28 wkGA | 26.0 (25.0, 27.0) |
| >28-34 wkGA | 33.0 (32.0, 34.0) |
| >34 wkGA | 37.0 (36.0, 38.0) |
| **RCG measurement timing** **for each time interval (wkGA)** |  |
| ≤20 wkGA | 10.0 (9.0, 13.0) |
| >20-28 wkGA | 25.0 (24.0, 26.0) |
| >28-34 wkGA | 32.0 (30.0, 33.0) |
| >34 wkGA | 37.0 (36.0, 38.0) |

1. **RCG measurements for each time interval (times)**

|  | **≤20 wkGA** | **>20-28 wkGA** | **>28-34 wkGA** | **>34 wkGA** |
| --- | --- | --- | --- | --- |
| **1** | 59,680 (78.29%) | 48,369 (63.45%) | 35,394 (46.43%) | 50,539 (66.30%) |
| **2** | 10,926 (14.33%) | 23,333 (30.61%) | 32,717 (42.92%) | 16,006 (21.00%) |
| **≥3** | 1,172 (1.54%) | 1,527 (2.00%) | 4,116 (5.40%) | 2,481 (3.25%) |
| **Missing** | 4,450 (5.84%) | 2,999 (3.93%) | 4,001 (5.25%) | 7,202 (9.45%) |

Table S2. Testing hour-standardized RCG z-scores and original RCG values (mmol/l)

| **Time** | **-3 SD** | **-2 SD** | **-1 SD** | **0 SD** | **+1 SD** | **+2 SD** | **+3 SD** |
| --- | --- | --- | --- | --- | --- | --- | --- |
| **Original values of RCG (mmol/L)** |  |  |  |  |  |  |  |
| ***Time of measurements*** |  |  |  |  |  |  |  |
| 7:00 | 2.40 | 3.49 | 4.58 | 5.66 | 6.75 | 7.84 | 8.93 |
| 8:00 | 2.38 | 3.51 | 4.65 | 5.79 | 6.93 | 8.07 | 9.20 |
| 9:00 | 2.27 | 3.39 | 4.52 | 5.65 | 6.78 | 7.90 | 9.03 |
| 10:00 | 2.16 | 3.29 | 4.42 | 5.55 | 6.68 | 7.81 | 8.94 |
| 11:00 | 2.10 | 3.22 | 4.34 | 5.45 | 6.57 | 7.68 | 8.79 |
| 12:00 | 1.99 | 3.13 | 4.27 | 5.42 | 6.56 | 7.70 | 8.84 |
| 13:00 | 2.31 | 3.44 | 4.58 | 5.72 | 6.86 | 8.00 | 9.13 |
| 14:00 | 2.39 | 3.50 | 4.61 | 5.72 | 6.83 | 7.94 | 9.05 |
| 15:00 | 2.46 | 3.53 | 4.60 | 5.67 | 6.74 | 7.81 | 8.87 |
| 16:00 | 2.42 | 3.47 | 4.52 | 5.56 | 6.61 | 7.66 | 8.71 |
| 17:00 | 2.35 | 3.39 | 4.43 | 5.46 | 6.50 | 7.54 | 8.58 |
| 18:00 | 2.35 | 3.39 | 4.43 | 5.46 | 6.50 | 7.54 | 8.58 |

***Table visualization***

Table S3. Diagnostic codes and register databases used to ascertain diagnoses within the Stockholm Youth Cohort (SYC).

|  | **Information on the mother of the index child/the index child** | **Variable** | **ICD-10** | **ATC** |
| --- | --- | --- | --- | --- |
| **Registry of Total Population (1968-2016)**  **Cause of Death (1961-2016)**  **Multi-generation Registry (as of 2010)** | Mother &Child  Child  Mother &Child  Mother &Child  Mother | Birthyear  Time resident in Sweden  Identity of biological mother and father  Sex  Maternal Migration Status (birth country) |  |  |
| **LISA^a^ (1990-2016)** | Mother | Maternal education |  |  |
| **National Patient Register**   - Inpatient Care (1973-2016) - Outpatient Specialist Care (2006-2016) - Psychiatric Outpatient (2006-2016)   **VAL register (1997-2016) ^b^**  **Stockholm Clinical Database for Child and Adolescent Psychiatry (2001-2016)** ^c^  **Medical Birth Register (1973-2010)** ^d^ | Child | Autism Spectrum Disorders (autism)^g^ | F84 |  |
|  | Child | Attention Deficit/Hyperactivity Disorder (ADHD)^g^ | F90 |  |
|  | Child | Intellectual Disability (ID)^g^ | F70-F79  F70: Mild (70>IQ≥50)  F71: Moderate (50>IQ≥35)  F72: Severe (35>IQ≥20)  F73: Profound (IQ<20)  F78/F79: Other/unspecified |  |
|  | Mother | Maternal psychiatric history | F chapter |  |
|  | Mother | Gestational hypertensive diseases | O13, O14, O15 |  |
|  | Child | Neonatal hypoglycemia | P70.3, P70.4 |  |
|  | Child | Neonatal birth trauma | P10-P15 |  |
|  | Mother | Long labour time | O63.0: Prolonged first stage;  O63.1: Prolonged second stage;  O63.9: Long labour, unspecified |  |
|  | Mother | Obstructed labour | O66.0: Obstructed labour due to shoulder dystocia;  O66.2: Obstructed labour due to unusually large fetus;  O66.4: Failed trial of labour, unspecified;  O66.5: Failed application of vacuum extractor and forceps, unspecified;  O66.8: Other specified obstructed labour;  O66.9: Obstructed labour, unspecified |  |
|  | Child | Congenital malformations, deformations, and chromosomal abnormalities | Q90-99 (all)  Q85.0  Q85.1 |  |
|  | Child | Disorders of amino acid metabolism | E70-E72 |  |
|  | Mother  Mother  Mother  Mother  Child  Child  Mother  Child | Maternal Height  Maternal Weight (first antenatal visit)  Number of gestations  Parity  Birth Weight ^f^  Gestational Week at Birth  Mode of delivery  Apgar Score |  |  |
| **Prescription Drug Register (2005-2016) ^\|^e** | Mother | Antidiabetic treatment |  | Glibenclamide/Glyburide [A10BB01]; Metformin [A10BA02]; Insulin [A10AB, A10AC, A10AD, A10AE, A10AF] |
|  | Child | Attention Deficit/Hyperactivity Disorder (ADHD) |  | methylphenidate [N06BA04] or atomoxetin [N06BA09] |

**Notes:**

Abbreviations: ADHD = Attention-Deficit/Hyperactivity Disorder; ID = Intellectual Disability; IQ = Intelligence Quotient; GDM= Gestational Diabetes Mellitus**;** ICD = International Classification of Diseases; ATC=Anatomical Therapeutic Chemical.

^a^ Longitudinal integrated database for health insurance and labour market studies.

^b^ VAL register: Stockholm county health care databases including in- and outpatient care, regardless of specialty/primary health care (coded according to ICD 10 since 2006).

^c^ Child and adolescent psychiatric in – and outpatient care within Stockholm County, including diagnosis and ratings of general functioning according to the Children's Global Assessment Scale. Diagnoses are coded according to DSM-IV groupings until 2008, and according to ICD-10 since 2009.

^d^ The Medical Birth Register (MBR): including information from medical records from prenatal, delivery, and neonatal healthcare beginning in 1973.

^e^ The Prescription Drug Register (PDR) contains data on medications dispensed to the entire population in Sweden since 1 July 2005. Receipt of a prescription for ADHD medications is a useful proxy for an ADHD diagnosis, as Swedish medical guidelines mandate that ADHD medications should only be prescribed by a psychiatric specialist and after other (non-pharmacological) interventions have failed.

^f^ Size for gestational age was calculated based on birthweight and gestational days at birth. **For male: Mean (birthweight)** = (-(1.907345*10^(-6))*days of gestation^4 + (1.140644*10^(-3))*days of gestation^3 -0.1336265*days^2 +1.976961*days of gestation+241.0053) **z-score**= (birthweight – mean [birthweight])/(0.12 * mean [birthweight]) **For female: Mean (birthweight)**= (-(2.761948*10^(-6))* days of gestation ^4 + (1.744841*10^(-3))* days of gestation s^3 -0.2893623* days of gestation^2 + 18.91197* days of gestation-413.5122);

**Z-score**= (birthweight – mean [birthweight])/(0.12 * mean [birthweight])

**Small for gestational age (SGA)**: z-score≤-2; **Large for gestational age (LGA)**: z-score≥2

^g^ As previously described in detail in our paper introducing the Stockholm Youth Cohort (Ref 37 in the main text): All services for children with NDCs, including diagnosis and follow-up health, special educational and social care, are provided by services run by, or contracted by, Stockholm Country and are available free of charge. Referrals for diagnostic evaluation of suspected neurodevelopmental conditions are commonly made by child healthcare centres, whose health- and developmental surveillance program engages 99.8% of all preschool children. Developmental surveillance is performed by specially trained child healthcare centre nurses at regular intervals (1, 2, 6, 10–12, 18, 36, 48 and 60 months of age), with examination by a pediatrician at key ages (2, 6, 10–12 months) and in case of developmental deviation or according to need at other age intervals. Speech abilities and language comprehension are evaluated by nurses at 36 and 48 months, and examination of sight and hearing is made at 48 months. The purpose of developmental surveillance is to ensure timely discovery of developmental problems such as autism, ID and ADHD. Referrals for diagnostic evaluation of suspected neurodevelopmental may also be requested by parents through general practitioners, pediatricians, child psychiatrists, speech therapists, or by schools as well as other health or social care agencies. Diagnostic evaluations are made by multi-professional teams, typically consisting of at least a psychologist and a medical doctor at child pediatric or mental health services.

**Table S4.** The details of the three best models (with the highest BIC values) for each group-based trajectory model with different numbers of groups.

|  | **Polynomials** | **Group size** | **BIC (panels)** | **Entropy** |
| --- | --- | --- | --- | --- |
| **1-Group GBTM** |  |  |  |  |
| GBTM 1 | 1 | 76228 | -377955.25 | NA |
| GBTM 2 | 3 | 76228 | -377753.87 | NA |
| GBTM 3 | 2 | 76228 | -377748.29 | NA |
| **2-Group GBTM** |  |  |  |  |
| GBTM 1 | 22 | 65416/10812 | -370553.25 | 0.676 |
| GBTM 2 | 23 | 65379/10849 | -370537.81 | 0.676 |
| GBTM 3 | 33 | 65306/10922 | -370533.71 | 0.674 |
| **3-Group GBTM** |  |  |  |  |
| GBTM 1 | 331 | 63664/9962/2602 | -369013.65 | 0.736 |
| GBTM 2 | 133 | 62837/11304/2087 | -368863.90 | 0.744 |
| GBTM 3 | 223 | 63317/10730/2181 | -368739.07 | 0.748 |
| **4-Group GBTM** |  |  |  |  |
| GBTM 1 | 2133 | 61013/11132/2652/1431 | -367288.67 | 0.759 |
| GBTM 2 | 3333 | 54656/17782/2706/1084 | -367285.85 | 0.694 |
| GBTM 3 | 3332 | 54719/17727/2706/1076 | -367281.43 | 0.695 |
| **5-Group GBTM** |  |  |  |  |
| GBTM 1 | 33233 | 17297/1057/53257/3192/1425 | -365904.32 | 0.718 |
| GBTM 2 | 33133 | 53324/1053/17048/3095/1708 | -365898.81 | 0.716 |
| GBTM 3 | 33333 | 53164/17319/1461/3178/1106 | -365882.02 | 0.716 |
| **6-Group GBTM** |  |  |  |  |
| GBTM 1 | 332333 | 51580/17097/899/1767/3559/1326 | -364873.99 | 0.726 |
| GBTM 2 | 333323 | 51581/17096/1326/1766/899/3560 | -364873.99 | 0.726 |
| GBTM 3 | 333333 | 1323/1722/51121/957/17538/3567 | -364849.65 | 0.723 |

**Table S5.** The model adequacy of the 5-group and the 6-group model.

| **5-Group cubic model (Entropy=0.716)** | | | | | | |
| --- | --- | --- | --- | --- | --- | --- |
| **Groups** | **N** | **The average posterior probability** | **The odds of correct classification (based on the max post prob group assignment)** | **The odds of correct classification (based on the weighted post prob group assignment)** | **Estimated group**  **probability** | **Proportions assigned to the group based on the posterior probability of group membership** |
| Group 1 | 53164 | .8551 | 2.5609 | 3.1881 | .6974 | .6493 |
| Group 2 | 17319 | .7111 | 8.3716 | 7.1402 | .2272 | .2563 |
| Group 3 | 3178 | .7288 | 61.7628 | 48.6522 | .0417 | .0523 |
| Group 4 | 1461 | .7277 | 136.7699 | 106.3250 | .0192 | .0245 |
| Group 5 | 1106 | .7935 | 261.0015 | 215.9566 | .0145 | .0175 |
|  | | |  |  |  |  |
| **6-Group cubic model (Entropy=0.723)** | | | | | | |
| Group 1 | 51121 | .8477 | 2.7377 | 3.3778 | .6706 | .6223 |
| Group 2 | 17538 | .6967 | 7.6868 | 6.7238 | .2301 | .2546 |
| Group 3 | 3567 | .7298 | 55.0242 | 44.5471 | .0458 | .0572 |
| Group 4 | 1722 | .7237 | 113.3019 | 88.8097 | .0226 | .0286 |
| Group 5 | 1323 | .7207 | 146.0616 | 112.5181 | .0174 | .0224 |
| Group 6 | 967 | .7298 | 265.2135 | 224.4509 | .0126 | .0148 |

**Table S6.** Characteristics of offspring whose mothers had no missing values in random capillary glucose level, with 1 missing value, or with more than 1 missing values in different time intervals (≤20, >20-28, >28-34, >34 wkGA) during pregnancy.

|  | **Included** | | **Excluded** | **P-value** |
| --- | --- | --- | --- | --- |
|  | **No missing** | **Missing in 1 time interval** | **Missing in >1 time intervals** |  |
| **Total** | 57576 | 18652 | 7047 | <0.001 |
| **Any NDCs, n (%)** | 2099 (3.6%) | 791 (4.2%) | 387 (5.5%) | <0.001 |
| **Maternal age, mean (SD)** | 31.3 (5.0) | 31.4 (5.1) | 30.6 (5.2) | <0.001 |
| **Maternal BMI, n (%)** |  |  |  |  |
| Normal | 38044 (66.1%) | 12325 (66.1%) | 4398 (62.4%) | <0.001 |
| Underweight | 1643 (2.9%) | 572 (3.1%) | 211 (3.0%) |  |
| Overweight | 12305 (21.4%) | 3767 (20.2%) | 1485 (21.1%) |  |
| Obese | 4629 (8.0%) | 1529 (8.2%) | 646 (9.2%) |  |
| *Missing* | 955 (1.7%) | 459 (2.5%) | 307 (4.4%) |  |
| **Maternal birth region, n (%)** |  |  |  |  |
| Nordic | 42836 (74.4%) | 13681 (73.3%) | 4889 (69.4%) | <0.001 |
| Europe | 3431 (6.0%) | 1136 (6.1%) | 467 (6.6%) |  |
| Africa | 2690 (4.7%) | 1022 (5.5%) | 498 (7.1%) |  |
| Asia | 7064 (12.3%) | 2267 (12.2%) | 953 (13.5%) |  |
| Other | 1549 (2.7%) | 545 (2.9%) | 240 (3.4%) |  |
| *Missing* | 6 (<1%) | <5 (<1%) | 0 (0.0%) |  |
| **Maternal psychiatric history, n (%)** |  |  |  |  |
| No | 51615 (89.6%) | 16715 (89.6%) | 6325 (89.8%) | 0.95 |
| Yes | 5961 (10.4%) | 1937 (10.4%) | 722 (10.2%) |  |
| **Maternal education level, n (%)** |  |  |  |  |
| Pre-high school | 5924 (10.3%) | 2142 (11.5%) | 967 (13.7%) | <0.001 |
| High school | 18597 (32.3%) | 5887 (31.6%) | 2250 (31.9%) |  |
| Post high school | 32744 (56.9%) | 10488 (56.2%) | 3750 (53.2%) |  |
| *Missing* | 311 (0.5%) | 135 (0.7%) | 80 (1.1%) |  |

**Abbreviations:** NDC, neurodevelopmental conditions; SD-standard deviation; BMI-body mass index

**Table S7.** Characteristics of offspring whose mothers had missing values in random capillary glucose levels during ≤20, >20-28, >28-34, and >34 wkGA throughout pregnancy.

|  | **≤20 wkGA** | |  | **>20-28 wkGA** | |  | **>28-34 wkGA** | |  | **>34 wkGA** | |  |
| --- | --- | --- | --- | --- | --- | --- | --- | --- | --- | --- | --- | --- |
|  | **No missing** | **Missing** | **P value** | **No missing** | **Missing** | **P value** | **No missing** | **Missing** | **P value** | **No missing** | **Missing** | **P value** |
| **Total** | 71778 | 4450 |  | 73229 | 2999 |  | 72227 | 4001 |  | 69026 | 7202 |  |
| **Maternal characteristics** |  |  |  |  |  |  |  |  |  |  |  |  |
| **Maternal age, mean (SD)** | 31.4 (5.0) | 30.8 (5.2) | <0.001 | 31.3 (5.1) | 31.4 (5.1) | 0.46 | 31.3 (5.1) | 31.9 (4.9) | <0.001 | 31.3 (5.0) | 31.4 (5.2) | 0.14 |
| **Maternal BMI, n (%)** |  |  |  |  |  |  |  |  |  |  |  |  |
| Normal | 47529 (66.2%) | 2840 (63.8%) | 0.017 | 48338 (66.0%) | 2031 (67.7%) | 0.067 | 47623 (65.9%) | 2746 (68.6%) | <0.001 | 45661 (66.2%) | 4708 (65.4%) | <0.001 |
| Underweight | 2105 (2.9%) | 110 (2.5%) |  | 2120 (2.9%) | 95 (3.2%) |  | 2090 (2.9%) | 125 (3.1%) |  | 1973 (2.9%) | 242 (3.4%) |  |
| Overweight | 15082 (21.0%) | 990 (22.2%) |  | 15481 (21.1%) | 591 (19.7%) |  | 15313 (21.2%) | 759 (19.0%) |  | 14645 (21.2%) | 1427 (19.8%) |  |
| Obese | 5781 (8.1%) | 377 (8.5%) |  | 5939 (8.1%) | 219 (7.3%) |  | 5880 (8.1%) | 278 (6.9%) |  | 5503 (8.0%) | 655 (9.1%) |  |
| *Missing* | 1281 (1.8%) | 133 (3.0%) |  | 1351 (1.8%) | 63 (2.1%) |  | 1321 (1.8%) | 93 (2.3%) |  | 1244 (1.8%) | 170 (2.4%) |  |
| **Maternal birth region, n (%)** |  |  |  |  |  |  |  |  |  |  |  |  |
| Nordic | 53349 (74.3%) | 3168 (71.2%) | <0.001 | 54385 (74.3%) | 2132 (71.1%) | <0.001 | 53443 (74.0%) | 3074 (76.8%) | <0.001 | 51210 (74.2%) | 5307 (73.7%) | 0.25 |
| Europe | 4260 (5.9%) | 307 (6.9%) |  | 4372 (6.0%) | 195 (6.5%) |  | 4343 (6.0%) | 224 (5.6%) |  | 4157 (6.0%) | 410 (5.7%) |  |
| Africa | 3423 (4.8%) | 289 (6.5%) |  | 3519 (4.8%) | 193 (6.4%) |  | 3524 (4.9%) | 188 (4.7%) |  | 3360 (4.9%) | 352 (4.9%) |  |
| Asia | 8780 (12.2%) | 551 (12.4%) |  | 8945 (12.2%) | 386 (12.9%) |  | 8939 (12.4%) | 392 (9.8%) |  | 8393 (12.2%) | 938 (13.0%) |  |
| Other | 1960 (2.7%) | 134 (3.0%) |  | 2001 (2.7%) | 93 (3.1%) |  | 1971 (2.7%) | 123 (3.1%) |  | 1899 (2.8%) | 195 (2.7%) |  |
| *Missing* | 6 (<1%) | <5 (<1%) |  | 7 (<1%) | 0 (0.0%) |  | 7 (<1%) | 0 (0.0%) |  | 7 (<1%) | 0 (0.0%) |  |
| **Maternal psychiatric history, n (%)** | 7554 (10.5%) | 344 (7.7%) | <0.001 | 7559 (10.3%) | 339 (11.3%) | 0.084 | 7508 (10.4%) | 390 (9.7%) | 0.19 | 7034 (10.2%) | 864 (12.0%) | <0.001 |
| **Maternal education level, n (%)** |  |  |  |  |  |  |  |  |  |  |  |  |
| Pre-high school | 7504 (10.5%) | 562 (12.6%) | <0.001 | 7698 (10.5%) | 368 (12.3%) | 0.005 | 7670 (10.6%) | 396 (9.9%) | 0.076 | 7250 (10.5%) | 816 (11.3%) | 0.034 |
| High school | 23106 (32.2%) | 1378 (31.0%) |  | 23565 (32.2%) | 919 (30.6%) |  | 23241 (32.2%) | 1243 (31.1%) |  | 22137 (32.1%) | 2347 (32.6%) |  |
| Post-high school | 40766 (56.8%) | 2466 (55.4%) |  | 41540 (56.7%) | 1692 (56.4%) |  | 40900 (56.6%) | 2332 (58.3%) |  | 39234 (56.8%) | 3998 (55.5%) |  |
| *Missing* | 402 (0.6%) | 44 (1.0%) |  | 426 (0.6%) | 20 (0.7%) |  | 416 (0.6%) | 30 (0.7%) |  | 405 (0.6%) | 41 (0.6%) |  |
| **GDM, n (%)** | 206 (0.3%) | 13 (0.3%) | 0.95 | 218 (0.3%) | <5 (<1%) | 0.008 | 209 (0.3%) | 10 (0.2%) | 0.65 | 152 (0.2%) | 67 (0.9%) | <0.001 |
|  |  |  |  |  |  |  |  |  |  |  |  |  |
| **Offspring characteristics** |  |  |  |  |  |  |  |  |  |  |  |  |
| **Gestational week at birth, n (%)** |  |  |  |  |  |  |  |  |  |  |  |  |
| Preterm | 2506 (3.5%) | 38 (0.9%) | <0.001 | 2512 (3.4%) | 32 (1.1%) | <0.001 | 2492 (3.5%) | 52 (1.3%) | <0.001 | 646 (0.9%) | **1898 (26.4%)** | <0.001 |
| Term | 64814 (90.3%) | 4056 (91.1%) |  | 66126 (90.3%) | 2744 (91.5%) |  | 65188 (90.3%) | 3682 (92.0%) |  | 63733 (92.3%) | 5137 (71.3%) |  |
| Post-term | 4458 (6.2%) | 356 (8.0%) |  | 4591 (6.3%) | 223 (7.4%) |  | 4547 (6.3%) | 267 (6.7%) |  | 4647 (6.7%) | 167 (2.3%) |  |
| **Size for gestational age, n (%)** |  |  |  |  |  |  |  |  |  |  |  |  |
| AGA | 68151 (94.9%) | 4231 (95.1%) | 0.47 | 69517 (94.9%) | 2865 (95.5%) | 0.17 | 68557 (94.9%) | 3825 (95.6%) | 0.18 | 65709 (95.2%) | 6673 (92.7%) | <0.001 |
| SGA | 1402 (2.0%) | 91 (2.0%) |  | 1448 (2.0%) | 45 (1.5%) |  | 1422 (2.0%) | 71 (1.8%) |  | 1197 (1.7%) | **296 (4.1%)** |  |
| LGA | 2106 (2.9%) | 117 (2.6%) |  | 2138 (2.9%) | 85 (2.8%) |  | 2123 (2.9%) | 100 (2.5%) |  | 2009 (2.9%) | **214 (3.0%)** |  |
| *Missing* | 119 (0.2%) | 11 (0.2%) |  | 126 (0.2%) | <5 (0.1%) |  | 125 (0.2%) | 5 (0.1%) |  | 111 (0.2%) | 19 (0.3%) |  |
| **Apgar score at 5-min <7, n (%)** |  |  |  |  |  |  |  |  |  |  |  |  |
| Yes | 476 (0.7%) | 27 (0.6%) | 0.66 | 484 (0.7%) | 19 (0.6%) | 0.85 | 477 (0.7%) | 26 (0.6%) | 0.93 | 424 (0.6%) | **79 (1.1%)** | <0.001 |
| *Missing* | 223 (0.3%) | 18 (0.4%) |  | 235 (0.3%) | 6 (0.2%) |  | 232 (0.3%) | 9 (0.2%) |  | 216 (0.3%) | 25 (0.3%) |  |
| **Any NDCs** | 2683 (3.7%) | **207 (4.7%)** | 0.002 | 2779 (3.8%) | 111 (3.7%) | 0.79 | 2742 (3.8%) | 148 (3.7%) | 0.75 | 2565 (3.7%) | **325 (4.5%)** | <0.001 |

**Abbreviations:** wkGA, weeks of gestational age; GDM, gestational diabetes mellitus; ID, intellectual disability; ADHD, attention-deficit hyperactivity; NDC, neurodevelopmental conditions; AGA, appropriate for gestational age; SGA, small for gestational age; LGA, large for gestational age.

**Table S8.** Characteristics of the study sample (N=76,228)

|  | **No NDCs (n=73338)** | **Any NDCs (n=2890)** | **P-value** |
| --- | --- | --- | --- |
| **Covariates** |  |  |  |
| **Child’s sex, n (%)** |  |  |  |
| Female | 36230 (49.4%) | 717 (24.8%) | <0.001 |
| Male | 37108 (50.6%) | 2173 (75.2%) |  |
| **Mother's age at delivery, mean (SD)** | 31.4 (5.0) | 30.1 (5.5) | <0.001 |
| **Maternal BMI, n (%)** |  |  |  |
| Normal weight (18.5 – 24.9 kg/m2) | 48690 (66.4%) | 1679 (58.1%) | <0.001 |
| Underweight (<18.5 kg/m2) | 2127 (2.9%) | 88 (3.0%) |  |
| Overweight (25.0 – 29.9 kg/m2) | 15375 (21.0%) | 697 (24.1%) |  |
| Obese (≥30 kg/m2) | 5797 (7.9%) | 361 (12.5%) |  |
| *Missing* | 1349 (1.8%) | 65 (2.2%) |  |
| **Maternal Birth Region, n (%)** | |  |  |
| Nordic | 54503 (74.3%) | 2014 (69.7%) | <0.001 |
| Europe | 4388 (6.0%) | 179 (6.2%) |  |
| Africa | 3532 (4.8%) | 180 (6.2%) |  |
| Asia | 8948 (12.2%) | 383 (13.3%) |  |
| Other | 1960 (2.7%) | 134 (4.6%) |  |
| *Missing* | 7 (<1%) | 0 (0.0%) |  |
| **Maternal psychiatric history, n (%)** | 7383 (10.1%) | 515 (17.8%) | <0.001 |
| **Maternal education level, n (%)** | |  | <0.001 |
| Pre-high school | 7551 (10.3%) | 515 (17.8%) |  |
| High school | 23326 (31.8%) | 1158 (40.1%) |  |
| Post-high school | 42033 (57.3%) | 1199 (41.5%) |  |
| *Missing* | 428 (0.6%) | 18 (0.6%) |  |
| **Parity, n (%)** |  |  |  |
| 1 | 33990 (46.3%) | 1500 (51.9%) | <0.001 |
| 2 | 27106 (37.0%) | 889 (30.8%) |  |
| ≥ 3 | 12242 (16.7%) | 501 (17.3%) |  |
| **GDM, n (%)** | 204 (0.3%) | 15 (0.5%) | 0.018 |
| **Antidiabetic treatment, n (%)** |  |  |  |
| No treatment | 73217 (99.8%) | 2877 (99.6%) | <0.001 |
| ≤20 wkGA | 49 (0.1%) | <5 (0.1%) |  |
| >20-28 wkGA | <5 (<1%) | <5 (0.1%) |  |
| >28-34 wkGA | 34 (<1%) | <5 (0.1%) |  |
| >34 wkGA | 34 (<1%) | 5 (0.2%) |  |
| **The earliest OGTT, n (%)** |  |  |  |
| No OGTT | 71310 (97.2%) | 2794 (96.7%) | 0.11 |
| ≤20 wkGA | 280 (0.4%) | 9 (0.3%) |  |
| >20-28 wkGA | 483 (0.7%) | 30 (1.0%) |  |
| >28-34 wkGA | 928 (1.3%) | 43 (1.5%) |  |
| >34 wkGA | 337 (0.5%) | 14 (0.5%) |  |
| **Gestational hypertensive diseases, n (%)** | 3564 (4.9%) | 178 (6.2%) | 0.002 |
| **Gestational week at birth, n (%)** | |  |  |
| Preterm (<37 weeks) | 2406 (3.3%) | 138 (4.8%) | <0.001 |
| Term (37-<42 weeks) | 66299 (90.4%) | 2571 (89.0%) |  |
| Post-term (≥42 weeks) | 4633 (6.3%) | 181 (6.3%) |  |
| **Size for gestational age, n (%)** | |  |  |
| AGA | 69690 (95.0%) | 2692 (93.1%) | <0.001 |
| SGA | 1393 (1.9%) | 100 (3.5%) |  |
| LGA | 2130 (2.9%) | 93 (3.2%) |  |
| *Missing* | 125 (0.2%) | 5 (0.2%) |  |
| **Macrosomia (birthweight>4500 g), n (%)** |  |  |  |
| Yes | 2238 (3.1%) | 90 (3.1%) | 0.85 |
| *Missing* | 125 (0.2%) | 5 (0.2%) |  |
| **Mode of delivery, n (%)** |  |  |  |
| Unassisted vaginal delivery | 45787 (62.4%) | 1630 (56.4%) | <0.001 |
| Induced vaginal delivery | 6702 (9.1%) | 344 (11.9%) |  |
| Assisted vaginal delivery | 6793 (9.3%) | 268 (9.3%) |  |
| Elective caesarean section | 7343 (10.0%) | 321 (11.1%) |  |
| Emergency caesarean section | 6713 (9.2%) | 327 (11.3%) |  |
| **Long labour duration, n (%)** | 1683 (2.3%) | 63 (2.2%) | 0.69 |
| **Obstructed labour, n (%)** | 678 (0.9%) | 26 (0.9%) | 0.89 |
| **Neonatal birth trauma, n (%)** | 1272 (1.7%) | 50 (1.7%) | 0.99 |
| **Apgar score at 5-min<7, n (%)** | |  |  |
| Yes | 470 (0.6%) | 33 (1.1%) | 0.001 |
| *Missing* | 235 (0.3%) | 6 (0.2%) |  |
| **Neonatal hypoglycemia, n (%)** | 1513 (2.1%) | 124 (4.3%) | <0.001 |

**Abbreviations:** wkGA, weeks of gestational age; GDM, gestational diabetes mellitus; ID, intellectual disability; ADHD, attention-deficit hyperactivity disorder; NDC, neurodevelopmental conditions; AGA, appropriate for gestational age; SGA, small for gestational age; LGA, large for gestational age; wkGA, weeks of gestational age; SD-standard deviation.

**Table S9.** Parameter estimation of the final trajectory model (with 5 cubic trajectories)

| Group |  | Parameter Estimate | Standard Error | T for H0: Parameter=0 | Prob>\|T\| |
| --- | --- | --- | --- | --- | --- |
| 1 | Intercept | -0.22433 | 0.03441 | -6.52 | <0.0001 |
|  | Linear | -0.17795 | 0.05252 | -3.389 | 0.0007 |
|  | Quadratic | 0.12568 | 0.02318 | 5.422 | <0.0001 |
|  | Cubic | -0.02301 | 0.00309 | -7.456 | <0.0001 |
|  |  |  |  |  |  |
| 2 | Intercept | -0.73455 | 0.07508 | -9.783 | <0.0001 |
|  | Linear | 1.0833 | 0.11531 | 9.395 | <0.0001 |
|  | Quadratic | -0.37017 | 0.05123 | -7.226 | <0.0001 |
|  | Cubic | 0.04797 | 0.00688 | 6.971 | <0.0001 |
|  |  |  |  |  |  |
| 3 | Intercept | 6.64887 | 0.16507 | 40.278 | <0.0001 |
|  | Linear | -7.01082 | 0.23906 | -29.326 | <0.0001 |
|  | Quadratic | 2.45625 | 0.10302 | 23.843 | <0.0001 |
|  | Cubic | -0.27979 | 0.01356 | -20.632 | <0.0001 |
|  |  |  |  |  |  |
| 4 | Intercept | -11.6706 | 0.34429 | -33.897 | <0.0001 |
|  | Linear | 18.56591 | 0.5357 | 34.657 | <0.0001 |
|  | Quadratic | -7.67846 | 0.2333 | -32.913 | <0.0001 |
|  | Cubic | 0.94134 | 0.03034 | 31.03 | <0.0001 |
|  |  |  |  |  |  |
| 5 | Intercept | 2.8553 | 0.31074 | 9.189 | <0.0001 |
|  | Linear | -4.06435 | 0.49301 | -8.244 | <0.0001 |
|  | Quadratic | 2.32617 | 0.22804 | 10.201 | <0.0001 |
|  | Cubic | -0.3452 | 0.03152 | -10.95 | <0.0001 |
|  |  |  |  |  |  |
|  | Sigma | 0.76225 | 0.00133 | 573.937 | <0.0001 |
|  |  |  |  |  |  |
| Group | membership | |  |  |  |
| 1 | (%) | 64.93146 | 0.65549 | 99.058 | <0.0001 |
| 2 | (%) | 25.63454 | 0.63879 | 40.13 | <0.0001 |
| 3 | (%) | 5.23373 | 0.1838 | 28.475 | <0.0001 |
| 4 | (%) | 2.45197 | 0.14796 | 16.572 | <0.0001 |
| 5 | (%) | 1.7483 | 0.10968 | 15.94 | <0.0001 |
| BIC=-365898.56 (N=286260) BIC=-365882.02 (N=76228) AIC=-365766.50 ll= -365741.50 | | | | | |
| Entropy = 0.716 | | | | | |

**Table S10.** The variance and covariance matrix of the final trajectory model (with 5 cubic trajectories)

|  | **Interc1** | **Linear1** | **Quadra1** | **Cubic1** | **Interc2** | **Linear2** | **Quadra2** | **Cubic2** | **Interc3** | **Linear3** | **Quadra3** | **Cubic3** | **Interc4** | **Linear4** | **Quadra4** | **Cubic4** | **Interc5** | **Linear5** | **Quadra5** | **Cubic5** | **Sigma** | **Theta2** | **Theta3** | **Theta4** | **Theta5** |
| --- | --- | --- | --- | --- | --- | --- | --- | --- | --- | --- | --- | --- | --- | --- | --- | --- | --- | --- | --- | --- | --- | --- | --- | --- | --- |
| **Interc1** | 0.001184 | -0.00178 | 0.000767 | -9.9E-05 | -0.00059 | 0.000899 | -0.0004 | 5.4E-05 | -3.8E-05 | 0.000215 | -0.00011 | 1.67E-05 | 0.000133 | -0.00035 | 0.000157 | -2.1E-05 | -0.00011 | 6.57E-05 | -1.7E-05 | -1.7E-07 | -5.3E-07 | 4.59E-05 | 0.000111 | -9.3E-05 | 0.000191 |
| **Linear1** | -0.00178 | 0.002758 | -0.00121 | 0.000159 | 0.000904 | -0.00139 | 0.000638 | -8.6E-05 | 0.000165 | -0.00042 | 0.000207 | -2.9E-05 | -0.00026 | 0.000624 | -0.00028 | 3.73E-05 | 0.000317 | -0.00035 | 0.00016 | -2E-05 | 3.14E-06 | -0.00015 | -0.00022 | 4.83E-05 | -0.00042 |
| **Quadra1** | 0.000767 | -0.00121 | 0.000537 | -7.1E-05 | -0.00041 | 0.000639 | -0.0003 | 4.08E-05 | -9.6E-05 | 0.000205 | -9.8E-05 | 1.35E-05 | 0.000156 | -0.00034 | 0.000148 | -2E-05 | -5.9E-05 | 7.89E-06 | 4.71E-06 | -2.7E-06 | -1.5E-06 | 4.96E-05 | 0.0001 | -1.2E-05 | 0.000185 |
| **Cubic1** | -9.9E-05 | 0.000159 | -7.1E-05 | 9.53E-06 | 5.41E-05 | -8.6E-05 | 4.07E-05 | -5.6E-06 | 1.42E-05 | -2.8E-05 | 1.34E-05 | -1.8E-06 | -2.4E-05 | 4.95E-05 | -2.2E-05 | 2.84E-06 | 3.1E-06 | 8.07E-06 | -5.2E-06 | 1.06E-06 | 2.22E-07 | -6.3E-06 | -1.4E-05 | 5.07E-07 | -2.6E-05 |
| **Interc2** | -0.00059 | 0.000904 | -0.00041 | 5.41E-05 | 0.005638 | -0.00852 | 0.003694 | -0.00048 | 0.000688 | -0.00031 | -6.1E-05 | 2.17E-05 | 0.003901 | -0.0064 | 0.002659 | -0.00032 | -0.00338 | 0.005856 | -0.00273 | 0.000382 | -1.8E-06 | 0.000189 | 0.001274 | -0.00048 | -0.00043 |
| **Linear2** | 0.000899 | -0.00139 | 0.000639 | -8.6E-05 | -0.00852 | 0.013295 | -0.00586 | 0.000773 | -0.00035 | -0.00036 | 0.000412 | -7.3E-05 | -0.00627 | 0.010175 | -0.0042 | 0.000502 | 0.00613 | -0.01083 | 0.005195 | -0.00074 | 7.54E-06 | -0.00047 | -0.00211 | 0.000432 | 0.0004 |
| **Quadra2** | -0.0004 | 0.000638 | -0.0003 | 4.07E-05 | 0.003694 | -0.00586 | 0.002624 | -0.00035 | 1.76E-05 | 0.000336 | -0.00026 | 4.21E-05 | 0.002367 | -0.00381 | 0.001548 | -0.00018 | -0.00279 | 0.005053 | -0.00245 | 0.000352 | -2.9E-06 | 0.000212 | 0.000842 | -0.00015 | -0.00026 |
| **Cubic2** | 5.4E-05 | -8.6E-05 | 4.08E-05 | -5.6E-06 | -0.00048 | 0.000773 | -0.00035 | 4.74E-05 | 2.41E-06 | -5.3E-05 | 3.83E-05 | -6.3E-06 | -0.00024 | 0.000382 | -0.00015 | 1.72E-05 | 0.000399 | -0.00073 | 0.000359 | -5.2E-05 | 3.01E-07 | -3.6E-05 | -9.7E-05 | 1.75E-05 | 3.69E-05 |
| **Interc3** | -3.8E-05 | 0.000165 | -9.6E-05 | 1.42E-05 | 0.000688 | -0.00035 | 1.76E-05 | 2.41E-06 | 0.02725 | -0.03867 | 0.016062 | -0.00203 | 0.006282 | -0.00808 | 0.003147 | -0.00038 | -0.00148 | 0.002143 | -0.00101 | 0.000149 | 1.62E-05 | 0.000471 | 0.000518 | -0.00261 | -0.00012 |
| **Linear3** | 0.000215 | -0.00042 | 0.000205 | -2.8E-05 | -0.00031 | -0.00036 | 0.000336 | -5.3E-05 | -0.03867 | 0.057152 | -0.02437 | 0.00314 | -0.00957 | 0.012459 | -0.00486 | 0.000586 | 0.002137 | -0.00312 | 0.001454 | -0.00021 | -1.4E-05 | -0.00052 | -0.00095 | 0.002741 | 0.000118 |
| **Quadra3** | -0.00011 | 0.000207 | -9.8E-05 | 1.34E-05 | -6.1E-05 | 0.000412 | -0.00026 | 3.83E-05 | 0.016062 | -0.02437 | 0.010613 | -0.00139 | 0.003807 | -0.00497 | 0.001933 | -0.00023 | -0.00101 | 0.001396 | -0.00062 | 8.69E-05 | 3.96E-06 | 0.000156 | 0.000403 | -0.00091 | -7.9E-05 |
| **Cubic3** | 1.67E-05 | -2.9E-05 | 1.35E-05 | -1.8E-06 | 2.17E-05 | -7.3E-05 | 4.21E-05 | -6.3E-06 | -0.00203 | 0.00314 | -0.00139 | 0.000184 | -0.00045 | 0.000583 | -0.00023 | 2.69E-05 | 0.000141 | -0.00019 | 8.09E-05 | -1.1E-05 | -3.7E-07 | -1.6E-05 | -5E-05 | 9.69E-05 | 1.21E-05 |
| **Interc4** | 0.000133 | -0.00026 | 0.000156 | -2.4E-05 | 0.003901 | -0.00627 | 0.002367 | -0.00024 | 0.006282 | -0.00957 | 0.003807 | -0.00045 | 0.118539 | -0.18312 | 0.078904 | -0.01013 | 0.008924 | -0.01491 | 0.006797 | -0.00089 | -7.3E-05 | -0.00178 | 0.014284 | -0.00026 | -0.00148 |
| **Linear4** | -0.00035 | 0.000624 | -0.00034 | 4.95E-05 | -0.0064 | 0.010175 | -0.00381 | 0.000382 | -0.00808 | 0.012459 | -0.00497 | 0.000583 | -0.18312 | 0.286972 | -0.12457 | 0.016078 | -0.01298 | 0.021458 | -0.00978 | 0.001291 | 0.000122 | 0.002921 | -0.02285 | 0.000186 | 0.00216 |
| **Quadra4** | 0.000157 | -0.00028 | 0.000148 | -2.2E-05 | 0.002659 | -0.0042 | 0.001548 | -0.00015 | 0.003147 | -0.00486 | 0.001933 | -0.00023 | 0.078904 | -0.12457 | 0.054428 | -0.00706 | 0.005787 | -0.00947 | 0.004279 | -0.00056 | -5.2E-05 | -0.00126 | 0.009746 | -5.7E-05 | -0.00098 |
| **Cubic4** | -2.1E-05 | 3.73E-05 | -2E-05 | 2.84E-06 | -0.00032 | 0.000502 | -0.00018 | 1.72E-05 | -0.00038 | 0.000586 | -0.00023 | 2.69E-05 | -0.01013 | 0.016078 | -0.00706 | 0.00092 | -0.00074 | 0.001208 | -0.00054 | 7.03E-05 | 6.59E-06 | 0.00016 | -0.00123 | 6.47E-06 | 0.000129 |
| **Interc5** | -0.00011 | 0.000317 | -5.9E-05 | 3.1E-06 | -0.00338 | 0.00613 | -0.00279 | 0.000399 | -0.00148 | 0.002137 | -0.00101 | 0.000141 | 0.008924 | -0.01298 | 0.005787 | -0.00074 | 0.096562 | -0.15105 | 0.068167 | -0.00917 | 2.24E-05 | -0.00287 | 0.000284 | -0.00088 | -0.00598 |
| **Linear5** | 6.57E-05 | -0.00035 | 7.89E-06 | 8.07E-06 | 0.005856 | -0.01083 | 0.005053 | -0.00073 | 0.002143 | -0.00312 | 0.001396 | -0.00019 | -0.01491 | 0.021458 | -0.00947 | 0.001208 | -0.15105 | 0.243056 | -0.11157 | 0.015178 | -3.8E-05 | 0.00494 | -0.00045 | 0.001457 | 0.009554 |
| **Quadra5** | -1.7E-05 | 0.00016 | 4.71E-06 | -5.2E-06 | -0.00273 | 0.005195 | -0.00245 | 0.000359 | -0.00101 | 0.001454 | -0.00062 | 8.09E-05 | 0.006797 | -0.00978 | 0.004279 | -0.00054 | 0.068167 | -0.11157 | 0.052004 | -0.00716 | 1.96E-05 | -0.00259 | 0.000154 | -0.00074 | -0.00492 |
| **Cubic5** | -1.7E-07 | -2E-05 | -2.7E-06 | 1.06E-06 | 0.000382 | -0.00074 | 0.000352 | -5.2E-05 | 0.000149 | -0.00021 | 8.69E-05 | -1.1E-05 | -0.00089 | 0.001291 | -0.00056 | 7.03E-05 | -0.00917 | 0.015178 | -0.00716 | 0.000994 | -2.7E-06 | 0.000389 | -1.6E-05 | 0.000107 | 0.000709 |
| **Sigma** | -5.3E-07 | 3.14E-06 | -1.5E-06 | 2.22E-07 | -1.8E-06 | 7.54E-06 | -2.9E-06 | 3.01E-07 | 1.62E-05 | -1.4E-05 | 3.96E-06 | -3.7E-07 | -7.3E-05 | 0.000122 | -5.2E-05 | 6.59E-06 | 2.24E-05 | -3.8E-05 | 1.96E-05 | -2.7E-06 | 1.76E-06 | -5.8E-06 | -2.5E-05 | -1.5E-05 | -1.9E-05 |
| **Theta2** | 4.59E-05 | -0.00015 | 4.96E-05 | -6.3E-06 | 0.000189 | -0.00047 | 0.000212 | -3.6E-05 | 0.000471 | -0.00052 | 0.000156 | -1.6E-05 | -0.00178 | 0.002921 | -0.00126 | 0.00016 | -0.00287 | 0.00494 | -0.00259 | 0.000389 | -5.8E-06 | 0.001184 | -3.8E-05 | 0.000197 | 0.001118 |
| **Theta3** | 0.000111 | -0.00022 | 0.0001 | -1.4E-05 | 0.001274 | -0.00211 | 0.000842 | -9.7E-05 | 0.000518 | -0.00095 | 0.000403 | -5E-05 | 0.014284 | -0.02285 | 0.009746 | -0.00123 | 0.000284 | -0.00045 | 0.000154 | -1.6E-05 | -2.5E-05 | -3.8E-05 | 0.00374 | 0.000237 | 0.000343 |
| **Theta4** | -9.3E-05 | 4.83E-05 | -1.2E-05 | 5.07E-07 | -0.00048 | 0.000432 | -0.00015 | 1.75E-05 | -0.00261 | 0.002741 | -0.00091 | 9.69E-05 | -0.00026 | 0.000186 | -5.7E-05 | 6.47E-06 | -0.00088 | 0.001457 | -0.00074 | 0.000107 | -1.5E-05 | 0.000197 | 0.000237 | 0.001408 | 0.000476 |
| **Theta5** | 0.000191 | -0.00042 | 0.000185 | -2.6E-05 | -0.00043 | 0.0004 | -0.00026 | 3.69E-05 | -0.00012 | 0.000118 | -7.9E-05 | 1.21E-05 | -0.00148 | 0.00216 | -0.00098 | 0.000129 | -0.00598 | 0.009554 | -0.00492 | 0.000709 | -1.9E-05 | 0.001118 | 0.000343 | 0.000476 | 0.004636 |

**Table S11.** Misclassification error matrix of the final trajectory model (with 5 cubic trajectories)

|  | **Group 1** | **Group 2** | **Group 3** | **Group 4** | **Group 5** |
| --- | --- | --- | --- | --- | --- |
| **Group 1** | .8551 | .1213 | .0181 | .0054 | .00003 |
| **Group 2** | .2031 | .7111 | .0374 | .0255 | .0229 |
| **Group 3** | .1245 | .1235 | .7288 | .0144 | .0089 |
| **Group 4** | .0808 | .1462 | .0262 | .7277 | .0192 |
| **Group 5** | .0016 | .1538 | .0247 | .0264 | .7935 |

**Table S12.** Statistics for model adequacy.

| **Full cohort, N=76,228 (Entropy=0.716)** | | | | | | |
| --- | --- | --- | --- | --- | --- | --- |
| **Groups** | **N** | **The average posterior probability** | **The odds of correct classification (based on the max post prob group assignment)** | **The odds of correct classification (based on the weighted post prob group assignment)** | **Estimated group**  **probability** | **Proportions assigned to the group based on the posterior probability of group membership** |
| Persistently Low | 53164 | .8551 | 2.5609 | 3.1881 | .6974 | .6493 |
| Moderate | 17319 | .7111 | 8.3716 | 7.1402 | .2272 | .2563 |
| High in Early Pregnancy | 3178 | .7288 | 61.7628 | 48.6522 | .0417 | .0523 |
| High in Mid-Pregnancy | 1461 | .7277 | 136.7699 | 106.3250 | .0192 | .0245 |
| Persistently High | 1106 | .7935 | 261.0015 | 215.9566 | .0145 | .0175 |
|  | | |  |  |  |  |
| **Replace the missing values with the smallest value during each stage of pregnancy, N=76,228 (Entropy=0.716)** | | | | | | |
| Persistently Low | 53165 | .8551 | 2.5608 | 3.1881 | .6974 | .6493 |
| Moderate | 17318 | .7111 | 8.3725 | 7.1405 | .2272 | .2563 |
| High in Early Pregnancy | 3178 | .7288 | 61.7648 | 48.6521 | .0417 | .0523 |
| High in Mid-Pregnancy | 1461 | .7277 | 136.7791 | 106.3269 | .0192 | .0245 |
| Persistently High | 1106 | .7935 | 260.9883 | 215.9509 | .0145 | .0175 |
|  |  |  |  |  |  |  |
| **Replace the missing values with the largest value during each stage of pregnancy, N=76,228 (Entropy=0.716)** | | | | | | |
| Persistently Low | 53165 | .8551 | 2.5608 | 3.1881 | .6974 | .6493 |
| Moderate | 17318 | .7111 | 8.3725 | 7.1405 | .2272 | .2563 |
| High in Early Pregnancy | 3178 | .7288 | 61.7648 | 48.6521 | .0417 | .0523 |
| High in Mid-Pregnancy | 1461 | .7277 | 136.7791 | 106.3268 | .0192 | .0245 |
| Persistently High | 1106 | .7935 | 260.9883 | 215.9509 | .0145 | .0175 |
|  |  |  |  |  |  |  |
| **Exclude the missing values during each stage of pregnancy, N_excluded_= 18,652 (Entropy=0.715)** | | | | | | |
| Persistently Low | 38535 | .8550 | 5.7667 | 6.5245 | .5055 | .4747 |
| Moderate | 14166 | .7154 | 11.0100 | 9.8049 | .1858 | .2040 |
| High in Early Pregnancy | 2570 | .7301 | 77.5347 | 65.5181 | .0337 | .0397 |
| High in Mid-Pregnancy | 1201 | .7213 | 161.6789 | 129.5177 | .0158 | .0196 |
| Persistently High | 1104 | .7752 | 234.6295 | 195.0871 | .0145 | .0174 |

**Table S13.** Sensitivity analysis by excluding women with overweight/obesity.

|  | **Full cohort (N=76,228)** | | | **Without overweight/obesity (N=52,584)** | | |
| --- | --- | --- | --- | --- | --- | --- |
|  | **OR (95% CI)** | **P-value** | **BH P-value^c^** | **OR (95% CI)** | **P-value** | **BH P-value^c^** |
| **Obstetric and neonatal outcomes^a^** |  |  |  |  |  |  |
| **Maternal outcomes** |  |  |  |  |  |  |
| **Gestational hypertensive diseases** |  |  |  |  |  |  |
| Group 1 (Persistently Low) | Ref. |  |  | Ref. |  |  |
| Group 2 (Moderate) | 0.98 (0.91-1.07) | 0.691 | 0.801 | 0.98 (0.87-1.09) | 0.665 | 0.872 |
| Group 3 (High in Early Pregnancy) | 1.08 (0.91-1.28) | 0.367 | 0.524 | 0.92 (0.72-1.18) | 0.511 | 0.728 |
| Group 4 (High in Mid-Pregnancy) | **1.48 (1.20-1.82)*** | <0.001 | <0.001 | 1.43 (1.06-1.94) | 0.019 | 0.084 |
| Group 5 (Persistently High) | **1.73 (1.40-2.15)*** | <0.001 | <0.001 | 1.38 (0.91-2.09) | 0.135 | 0.284 |
| **Induced vaginal delivery** |  |  |  |  |  |  |
| Group 1 (Persistently Low) | Ref. |  |  | Ref. |  |  |
| Group 2 (Moderate) | 1.01 (0.95-1.07) | 0.835 | 0.915 | 1.01 (0.93-1.09) | 0.894 | 0.933 |
| Group 3 (High in Early Pregnancy) | 1.06 (0.94-1.20) | 0.356 | 0.521 | 0.98 (0.83-1.15) | 0.779 | 0.890 |
| Group 4 (High in Mid-Pregnancy) | 1.11 (0.93-1.33) | 0.260 | 0.408 | 0.87 (0.67-1.12) | 0.279 | 0.483 |
| Group 5 (Persistently High) | **1.36 (1.12-1.66)*** | 0.002 | 0.008 | 1.40 (1.03-1.91) | 0.032 | 0.116 |
| **Assisted vaginal delivery** |  |  |  |  |  |  |
| Group 1 (Persistently Low) | Ref. |  |  | Ref. |  |  |
| Group 2 (Moderate) | **1.08 (1.02-1.15)*** | 0.013 | 0.044 | **1.11 (1.03-1.20)*** | 0.007 | 0.032 |
| Group 3 (High in Early Pregnancy) | 1.11 (0.97-1.28) | 0.123 | 0.223 | 1.17 (1.00-1.36) | 0.057 | 0.153 |
| Group 4 (High in Mid-Pregnancy) | 1.16 (0.95-1.40) | 0.142 | 0.252 | 1.06 (0.83-1.35) | 0.631 | 0.852 |
| Group 5 (Persistently High) | 1.27 (1.01-1.59) | 0.041 | 0.092 | 1.34 (0.98-1.83) | 0.071 | 0.169 |
| **Caesarean section** |  |  |  |  |  |  |
| Group 1 (Persistently Low) | Ref. |  |  | Ref. |  |  |
| Group 2 (Moderate) | **1.15 (1.10-1.20)*** | <0.001 | <0.001 | **1.15 (1.09-1.22)*** | <0.001 | <0.001 |
| Group 3 (High in Early Pregnancy) | 1.11 (1.01-1.21) | 0.029 | 0.073 | 1.07 (0.96-1.21) | 0.223 | 0.407 |
| Group 4 (High in Mid-Pregnancy) | **1.24 (1.09-1.40)*** | 0.001 | 0.004 | 1.19 (1.01-1.41) | 0.044 | 0.136 |
| Group 5 (Persistently High) | **1.48 (1.29-1.71)*** | <0.001 | <0.001 | **1.51 (1.21-1.88)*** | <0.001 | 0.002 |
| **Long labour time** |  |  |  |  |  |  |
| Group 1 (Persistently Low) | Ref. |  |  | Ref. |  |  |
| Group 2 (Moderate) | 1.14 (1.02-1.28) | 0.023 | 0.067 | 1.17 (1.02-1.35) | 0.027 | 0.105 |
| Group 3 (High in Early Pregnancy) | 1.05 (0.81-1.35) | 0.720 | 0.820 | 1.18 (0.87-1.59) | 0.284 | 0.483 |
| Group 4 (High in Mid-Pregnancy) | 1.16 (0.83-1.61) | 0.380 | 0.533 | 1.04 (0.67-1.62) | 0.864 | 0.933 |
| Group 5 (Persistently High) | 1.33 (0.94-1.88) | 0.105 | 0.200 | 1.56 (0.96-2.54) | 0.072 | 0.169 |
| **Obstructed labour** |  |  |  |  |  |  |
| Group 1 (Persistently Low) | Ref. |  |  | Ref. |  |  |
| Group 2 (Moderate) | **1.45 (1.22-1.71)*** | <0.001 | <0.001 | 1.29 (1.03-1.61) | 0.027 | 0.105 |
| Group 3 (High in Early Pregnancy) | 1.51 (1.07-2.13) | 0.018 | 0.057 | 1.26 (0.79-2.01) | 0.337 | 0.562 |
| Group 4 (High in Mid-Pregnancy) | 1.55 (0.97-2.47) | 0.066 | 0.134 | 1.89 (1.07-3.31) | 0.028 | 0.105 |
| Group 5 (Persistently High) | **2.46 (1.62-3.74)*** | <0.001 | <0.001 | **3.00 (1.66-5.43)*** | <0.001 | 0.002 |
|  |  |  |  |  |  |  |
| **Neonatal outcomes^a^** |  |  |  |  |  |  |
| **Preterm birth** |  |  |  |  |  |  |
| Group 1 (Persistently Low) | Ref. |  |  | Ref. |  |  |
| Group 2 (Moderate) | 0.90 (0.81-0.99) | 0.029 | 0.073 | 0.89 (0.78-1.01) | 0.067 | 0.169 |
| Group 3 (High in Early Pregnancy) | 1.13 (0.93-1.37) | 0.215 | 0.358 | 1.02 (0.79-1.31) | 0.885 | 0.933 |
| Group 4 (High in Mid-Pregnancy) | **1.38 (1.08-1.77)*** | 0.011 | 0.039 | 1.35 (0.98-1.88) | 0.069 | 0.169 |
| Group 5 (Persistently High) | **1.70 (1.32-2.20)*** | <0.001 | <0.001 | 1.44 (0.94-2.19) | 0.091 | 0.208 |
| **Small for gestational age** |  |  |  |  |  |  |
| Group 1 (Persistently Low) | Ref. |  |  | Ref. |  |  |
| Group 2 (Moderate) | **0.74 (0.65-0.85)** | <0.001 | <0.001 | **0.78 (0.67-0.92)*** | 0.002 | 0.014 |
| Group 3 (High in Early Pregnancy) | 1.10 (0.86-1.40) | 0.459 | 0.601 | 1.07 (0.80-1.44) | 0.639 | 0.852 |
| Group 4 (High in Mid-Pregnancy) | 0.94 (0.66-1.34) | 0.728 | 0.820 | 1.08 (0.71-1.63) | 0.732 | 0.890 |
| Group 5 (Persistently High) | 0.56 (0.34-0.92) | 0.023 | 0.067 | 0.48 (0.22-1.02) | 0.055 | 0.153 |
| **Large for gestational age** |  |  |  |  |  |  |
| Group 1 (Persistently Low) | Ref. |  |  | Ref. |  |  |
| Group 2 (Moderate) | **1.61 (1.46-1.77)*** | <0.001 | <0.001 | **1.56 (1.35-1.80)*** | <0.001 | <0.001 |
| Group 3 (High in Early Pregnancy) | 1.24 (1.01-1.53) | 0.042 | 0.092 | 1.26 (0.93-1.70) | 0.132 | 0.284 |
| Group 4 (High in Mid-Pregnancy) | **2.28 (1.79-2.89)*** | <0.001 | <0.001 | **1.99 (1.34-2.95)*** | 0.001 | 0.004 |
| Group 5 (Persistently High) | **4.01 (3.23-4.99)*** | <0.001 | <0.001 | **3.87 (2.56-5.87)*** | <0.001 | <0.001 |
| **Macrosomia** |  |  |  |  |  |  |
| Group 1 (Persistently Low) | Ref. |  |  | Ref. |  |  |
| Group 2 (Moderate) | **1.55 (1.41-1.71)*** | <0.001 | <0.001 | **1.52 (1.33-1.74)*** | <0.001 | <0.001 |
| Group 3 (High in Early Pregnancy) | **1.29 (1.05-1.57)*** | 0.014 | 0.044 | **1.47 (1.13-1.91)*** | 0.004 | 0.022 |
| Group 4 (High in Mid-Pregnancy) | **1.65 (1.26-2.15)*** | <0.001 | 0.001 | **1.76 (1.19-2.58)*** | 0.004 | 0.022 |
| Group 5 (Persistently High) | **2.73 (2.13-3.49)*** | <0.001 | <0.001 | **2.70 (1.73-4.23)*** | <0.001 | <0.001 |
| **Neonatal birth trauma** |  |  |  |  |  |  |
| Group 1 (Persistently Low) | Ref. |  |  | Ref. |  |  |
| Group 2 (Moderate) | **1.40 (1.23-1.58)*** | <0.001 | <0.001 | **1.53 (1.31-1.78)*** | <0.001 | <0.001 |
| Group 3 (High in Early Pregnancy) | **1.42 (1.10-1.83)*** | 0.007 | 0.026 | 1.38 (1.00-1.90) | 0.049 | 0.146 |
| Group 4 (High in Mid-Pregnancy) | 1.21 (0.82-1.77) | 0.335 | 0.514 | 1.03 (0.60-1.76) | 0.921 | 0.933 |
| Group 5 (Persistently High) | **1.67 (1.15-2.41)*** | 0.007 | 0.025 | 1.77 (1.03-3.05) | 0.039 | 0.131 |
| **Apgar score at 5-min <7** |  |  |  |  |  |  |
| Group 1 (Persistently Low) | Ref. |  |  | Ref. |  |  |
| Group 2 (Moderate) | 1.18 (0.96-1.45) | 0.112 | 0.208 | 1.18 (0.92-1.53) | 0.199 | 0.379 |
| Group 3 (High in Early Pregnancy) | 1.01 (0.64-1.59) | 0.969 | 0.981 | 0.89 (0.49-1.64) | 0.711 | 0.890 |
| Group 4 (High in Mid-Pregnancy) | 1.10 (0.60-2.01) | 0.767 | 0.852 | 0.89 (0.37-2.17) | 0.798 | 0.899 |
| Group 5 (Persistently High) | 1.35 (0.73-2.49) | 0.341 | 0.514 | 2.12 (0.98-4.56) | 0.056 | 0.153 |
| **Neonatal hypoglycemia** |  |  |  |  |  |  |
| Group 1 (Persistently Low) | Ref. |  |  | Ref. |  |  |
| Group 2 (Moderate) | **1.23 (1.09-1.38)*** | <0.001 | 0.002 | 1.19 (1.01-1.39) | 0.035 | 0.121 |
| Group 3 (High in Early Pregnancy) | **1.66 (1.35-2.05)*** | <0.001 | <0.001 | **1.71 (1.30-2.26)*** | <0.001 | 0.002 |
| Group 4 (High in Mid-Pregnancy) | 1.26 (0.91-1.75) | 0.160 | 0.278 | 1.25 (0.78-1.99) | 0.352 | 0.563 |
| Group 5 (Persistently High) | **2.68 (2.08-3.46)*** | <0.001 | <0.001 | **2.36 (1.50-3.71)*** | <0.001 | 0.002 |
|  |  |  |  |  |  |  |
| **Neurodevelopmental conditions^b^** |  |  |  |  |  |  |
| **Any NDCs** |  |  |  |  |  |  |
| Group 1 (Persistently Low) | Ref. |  |  | Ref. |  |  |
| Group 2 (Moderate) | 1.06 (0.97-1.16) | 0.215 | 0.358 | 1.08 (0.96-1.21) | 0.224 | 0.407 |
| Group 3 (High in Early Pregnancy) | 1.18 (0.98-1.41) | 0.078 | 0.156 | 1.26 (1.01-1.59) | 0.044 | 0.136 |
| Group 4 (High in Mid-Pregnancy) | 1.11 (0.85-1.43) | 0.447 | 0.596 | 0.96 (0.66-1.39) | 0.834 | 0.927 |
| Group 5 (Persistently High) | 1.08 (0.82-1.44) | 0.575 | 0.708 | 0.93 (0.57-1.52) | 0.764 | 0.890 |
| **Any autism** |  |  |  |  |  |  |
| Group 1 (Persistently Low) | Ref. |  |  | Ref. |  |  |
| Group 2 (Moderate) | 1.01 (0.89-1.14) | 0.899 | 0.959 | 1.06 (0.90-1.24) | 0.478 | 0.722 |
| Group 3 (High in Early Pregnancy) | 1.12 (0.87-1.44) | 0.389 | 0.537 | 1.27 (0.94-1.72) | 0.125 | 0.278 |
| Group 4 (High in Mid-Pregnancy) | 1.01 (0.70-1.46) | 0.950 | 0.974 | 0.93 (0.56-1.55) | 0.778 | 0.890 |
| Group 5 (Persistently High) | 0.76 (0.48-1.19) | 0.225 | 0.367 | 0.96 (0.51-1.81) | 0.900 | 0.933 |
| **Any ID** |  |  |  |  |  |  |
| Group 1 (Persistently Low) | Ref. |  |  | Ref. |  |  |
| Group 2 (Moderate) | 1.06 (0.84-1.34) | 0.619 | 0.728 | 1.10 (0.82-1.49) | 0.519 | 0.728 |
| Group 3 (High in Early Pregnancy) | 1.51 (1.01-2.26) | 0.047 | 0.101 | 1.13 (0.61-2.07) | 0.706 | 0.890 |
| Group 4 (High in Mid-Pregnancy) | 1.00 (0.51-1.96) | 0.992 | 0.992 | 1.05 (0.42-2.59) | 0.920 | 0.933 |
| Group 5 (Persistently High) | 0.59 (0.24-1.45) | 0.252 | 0.403 | 0.99 (0.31-3.13) | 0.982 | 0.982 |
| **Any ADHD** |  |  |  |  |  |  |
| Group 1 (Persistently Low) | Ref. |  |  | Ref. |  |  |
| Group 2 (Moderate) | 1.11 (0.99-1.24) | 0.086 | 0.168 | 1.11 (0.95-1.29) | 0.189 | 0.371 |
| Group 3 (High in Early Pregnancy) | 1.29 (1.03-1.62) | 0.029 | 0.073 | **1.51 (1.15-2.00)*** | 0.004 | 0.020 |
| Group 4 (High in Mid-Pregnancy) | 1.09 (0.78-1.53) | 0.610 | 0.728 | 0.80 (0.47-1.34) | 0.390 | 0.612 |
| Group 5 (Persistently High) | 1.37 (0.98-1.90) | 0.065 | 0.135 | 0.79 (0.39-1.60) | 0.512 | 0.728 |
| **Autism only** |  |  |  |  |  |  |
| Group 1 (Persistently Low) | Ref. |  |  | Ref. |  |  |
| Group 2 (Moderate) | 0.94 (0.80-1.11) | 0.475 | 0.603 | 0.99 (0.80-1.22) | 0.898 | 0.933 |
| Group 3 (High in Early Pregnancy) | 0.87 (0.60-1.26) | 0.466 | 0.601 | 0.93 (0.59-1.47) | 0.761 | 0.890 |
| Group 4 (High in Mid-Pregnancy) | 1.13 (0.71-1.79) | 0.616 | 0.728 | 1.20 (0.67-2.16) | 0.537 | 0.741 |
| Group 5 (Persistently High) | 0.81 (0.46-1.45) | 0.485 | 0.606 | 1.32 (0.65-2.68) | 0.445 | 0.685 |
| **ADHD only** |  |  |  |  |  |  |
| Group 1 (Persistently Low) | Ref. |  |  | Ref. |  |  |
| Group 2 (Moderate) | 1.16 (1.01-1.32) | 0.033 | 0.080 | 1.14 (0.96-1.37) | 0.142 | 0.291 |
| Group 3 (High in Early Pregnancy) | 1.12 (0.84-1.50) | 0.442 | 0.596 | 1.27 (0.89-1.82) | 0.190 | 0.371 |
| Group 4 (High in Mid-Pregnancy) | 1.20 (0.82-1.75) | 0.358 | 0.521 | 0.91 (0.50-1.63) | 0.740 | 0.890 |
| Group 5 (Persistently High) | 1.54 (1.06-2.24) | 0.024 | 0.067 | 0.74 (0.30-1.79) | 0.498 | 0.728 |
| **Autism and ADHD** |  |  |  |  |  |  |
| Group 1 (Persistently Low) | Ref. |  |  | Ref. |  |  |
| Group 2 (Moderate) | 1.02 (0.81-1.29) | 0.875 | 0.946 | 1.05 (0.78-1.42) | 0.758 | 0.890 |
| Group 3 (High in Early Pregnancy) | 1.56 (1.03-2.36) | 0.035 | 0.082 | **2.10 (1.32-3.34)*** | 0.002 | 0.012 |
| Group 4 (High in Mid-Pregnancy) | 0.96 (0.48-1.95) | 0.918 | 0.966 | 0.44 (0.11-1.77) | 0.248 | 0.441 |
| Group 5 (Persistently High) | 0.97 (0.45-2.09) | 0.947 | 0.974 | 0.40 (0.06-2.78) | 0.352 | 0.563 |

^a^ GEE model, clustered on maternal identification numbers. Adjusted for child’s sex and birthyear, maternal birth region, maternal age, maternal education level, maternal BMI, and parity.

^b^ GEE model, clustered on maternal identification numbers. Adjusted for covariates in “a” and maternal psychiatric history.

^c^ Benjamini-Hochberg (BH) FDR correction. After applying the BH FDR correction, results that remained significant are marked with an asterisk (*) and highlighted in black bold.

**Table S14.** Sensitivity analysis for the association between glucose trajectories and offspring NDCs by excluding gestational hypertensive diseases.

|  | **Full cohort (N=76,228)** | | **Without gestational hypertensive diseases (N=** **72,486)** | |
| --- | --- | --- | --- | --- |
|  | **OR (95% CI)^a^** | **P-value** | **OR (95% CI)^a^** | **P-value** |
| **Neurodevelopmental conditions** |  |  |  |  |
| **Any NDCs** |  |  |  |  |
| Group 1 (Persistently Low) | Ref. |  | Ref. |  |
| Group 2 (Moderate) | 1.06 (0.97-1.16) | 0.215 | 1.07 (0.98-1.17) | 0.156 |
| Group 3 (High in Early Pregnancy) | 1.18 (0.98-1.41) | 0.078 | 1.19 (0.99-1.44) | 0.069 |
| Group 4 (High in Mid-Pregnancy) | 1.11 (0.85-1.43) | 0.447 | 1.12 (0.86-1.47) | 0.402 |
| Group 5 (Persistently High) | 1.08 (0.82-1.44) | 0.575 | 1.05 (0.77-1.42) | 0.760 |
| **Any autism** |  |  |  |  |
| Group 1 (Persistently Low) | Ref. |  | Ref. |  |
| Group 2 (Moderate) | 1.01 (0.89-1.14) | 0.899 | 1.03 (0.91-1.17) | 0.636 |
| Group 3 (High in Early Pregnancy) | 1.12 (0.87-1.44) | 0.389 | 1.16 (0.89-1.50) | 0.265 |
| Group 4 (High in Mid-Pregnancy) | 1.01 (0.70-1.46) | 0.950 | 1.05 (0.72-1.54) | 0.796 |
| Group 5 (Persistently High) | 0.76 (0.48-1.19) | 0.225 | 0.74 (0.46-1.20) | 0.224 |
| **Any ID** |  |  |  |  |
| Group 1 (Persistently Low) | Ref. |  | Ref. |  |
| Group 2 (Moderate) | 1.06 (0.84-1.34) | 0.619 | 1.11 (0.87-1.41) | 0.400 |
| Group 3 (High in Early Pregnancy) | 1.51 (1.01-2.26) | 0.047 | 1.47 (0.96-2.27) | 0.079 |
| Group 4 (High in Mid-Pregnancy) | 1.00 (0.51-1.96) | 0.992 | 1.13 (0.58-2.22) | 0.714 |
| Group 5 (Persistently High) | 0.59 (0.24-1.45) | 0.252 | 0.55 (0.20-1.50) | 0.245 |
| **Any ADHD** |  |  |  |  |
| Group 1 (Persistently Low) | Ref. |  | Ref. |  |
| Group 2 (Moderate) | 1.11 (0.99-1.24) | 0.086 | 1.12 (1.00-1.26) | 0.053 |
| Group 3 (High in Early Pregnancy) | 1.29 (1.03-1.62) | 0.029 | 1.31 (1.03-1.65) | 0.026 |
| Group 4 (High in Mid-Pregnancy) | 1.09 (0.78-1.53) | 0.610 | 1.06 (0.75-1.52) | 0.730 |
| Group 5 (Persistently High) | 1.37 (0.98-1.90) | 0.065 | 1.24 (0.86-1.79) | 0.254 |
| **Autism only** |  |  |  |  |
| Group 1 (Persistently Low) | Ref. |  | Ref. |  |
| Group 2 (Moderate) | 0.94 (0.80-1.11) | 0.475 | 0.94 (0.79-1.11) | 0.459 |
| Group 3 (High in Early Pregnancy) | 0.87 (0.60-1.26) | 0.466 | 0.90 (0.62-1.32) | 0.596 |
| Group 4 (High in Mid-Pregnancy) | 1.13 (0.71-1.79) | 0.616 | 1.17 (0.73-1.89) | 0.516 |
| Group 5 (Persistently High) | 0.81 (0.46-1.45) | 0.485 | 0.91 (0.51-1.62) | 0.748 |
| **ADHD only** |  |  |  |  |
| Group 1 (Persistently Low) | Ref. |  | Ref. |  |
| Group 2 (Moderate) | 1.16 (1.01-1.32) | 0.033 | 1.16 (1.01-1.33) | 0.039 |
| Group 3 (High in Early Pregnancy) | 1.12 (0.84-1.50) | 0.442 | 1.15 (0.85-1.54) | 0.365 |
| Group 4 (High in Mid-Pregnancy) | 1.20 (0.82-1.75) | 0.358 | 1.15 (0.77-1.71) | 0.510 |
| Group 5 (Persistently High) | 1.54 (1.06-2.24) | 0.024 | 1.43 (0.95-2.14) | 0.088 |
| **Autism and ADHD** |  |  |  |  |
| Group 1 (Persistently Low) | Ref. |  | Ref. |  |
| Group 2 (Moderate) | 1.02 (0.81-1.29) | 0.875 | 1.05 (0.83-1.34) | 0.675 |
| Group 3 (High in Early Pregnancy) | 1.56 (1.03-2.36) | 0.035 | 1.58 (1.03-2.43) | 0.038 |
| Group 4 (High in Mid-Pregnancy) | 0.96 (0.48-1.95) | 0.918 | 0.95 (0.45-2.01) | 0.886 |
| Group 5 (Persistently High) | 0.97 (0.45-2.09) | 0.947 | 0.81 (0.33-1.98) | 0.648 |

^a^ GEE model, clustered on maternal identification numbers. Adjusted for child’s sex and birthyear, maternal birth region, maternal age, maternal education level, maternal BMI, parity, and maternal psychiatric history.
