# Supplementary material for: Random capillary glucose levels throughout pregnancy, obstetric and neonatal outcomes, and long-term neurodevelopmental conditions in children: a group-based trajectory analysis
Source: BMC Med. 2023 Jul 19;21:260. doi: 10.1186/s12916-023-02926-3 (PMC10354916; doi:10.1186/s12916-023-02926-3)
Supplement: Supplementary file 2 — Additional file 2. GRoLTS checklist. Guidelines for Reporting on Latent Trajectory Studieswere adhered to bolster our reporting on the Group-Based Trajectory Modeling. [file 12916_2023_2926_MOESM2_ESM.docx]

**Checklist:** Guidelines for Reporting on Latent Trajectory Studies (GRoLTS)

1. **Is the metric of time used in the statistical model reported?**

Yes.

See **Additional file 1-Supplementary methods** and **Table S1.**

1. **Is information presented about the mean and variance of time within a wave?**

Yes.

See **Additional file 1-Supplementary methods** and **Table S1**.

1. **(a) Is the missing data mechanism reported?**

Yes.

See **Methods-Statistical analysis,** and **Table S6.**

**(b) Is a description provided of what variables are related to attrition/missing data?**

Yes.

See **Additional file 1- Table S6-7**.

**(c) Is a description provided of how missing data in the analyses were dealt with?**

Yes.

See **Methods-Sensitivity analysis; Results-sensitivity analysis; Additional file 1-Fig S7; Table S12**.

1. **Is information about the distribution of the observed variables included?**

Yes.

See **Additional file 1-Fig S3**.

1. **Is the software mentioned?**

Yes.

See **Methods-Statistical analysis (first sentence)**.

1. **(a) Are alternative specifications of within-class** **heterogeneity considered (e.g., LGCA vs. LGMM) and clearly documented? If not, was sufficient justification provided as to eliminate certain specifications from consideration?**

Yes.

See **Fig 1C (Spaghetti plotting)** and **Discussion-methodology consideration for GBTM.**

**(b) Are alternative specifications of the between-class differences in variance–covariance matrix structure considered and clearly documented? If not, was sufficient justification provided as to eliminate certain specifications from consideration?**

See the variance-covariance matrix of the final sample in **Additional file 1-supplementary methods** and **Table S10**.

1. **Are alternative shape/functional forms of the trajectories described?**

Yes.

See **Additional file 1-Supplementary methods** and **Fig S6**.

1. **If covariates have been used, can analyses still be replicated?**

See **Discussion-Methodology considerations for GBTM.**

1. **Is information reported about the number of random start values and final iterations included?**

Yes.

See **Additional file 1-Supplementary methods** and **Methods-Statistical analysis**.

1. **Are the model comparison (and selection) tools described from a statistical perspective?**

Yes.

See **Additional file 1-Supplementary methods** and **Table S12**.

1. **Are the total number of fitted models reported, including a one-class solution?**

Yes.

See **Additional file 1-Supplementary methods** and **Table S4**.

1. **Are the number of cases per class reported for each model (absolute sample size, or proportion)?**

Yes.

See **Additional file 1-Supplementary methods, Table S4**, and **Table S12**.

1. **If classification of cases in a trajectory is the goal, is entropy reported?**

Yes.

See **Additional file 1-Supplementary methods** and **Table S12**.

1. **(a) Is a plot included with the estimated mean trajectories of the final solution?**

Yes.

See **Fig 1A**.

**(b) Are plots included with the estimated mean trajectories for each model?**

Yes.

See **Additional file 1-Supplementary methods** and **Fig S6**.

**(c)** **Is a plot included of the combination of estimated means of the final model and the observed individual trajectories split out for each latent class?**

Yes.

See **Fig 1A and 1C**.

1. **Are characteristics of the final class solution numerically described (i.e., means, SD/SE, n, CI, etc.)?**

Yes.

See **Additional file 1- Supplementary methods,** and **Table S9**.

1. **Are the syntax files available (either in the appendix, supplementary materials, or from the authors)?**

Available upon request to the corresponding author.
